# Atomic‐ and Molecular‐Level Design of Functional Metal–Organic Frameworks (MOFs) and Derivatives for Energy and Environmental Applications

**DOI:** 10.1002/advs.201901129

**Published:** 2019-09-01

**Authors:** Gamze Yilmaz, Shing Bo Peh, Dan Zhao, Ghim Wei Ho

**Affiliations:** ^1^ Department of Electrical and Computer Engineering National University of Singapore 4 Engineering Drive 3 Singapore 117583 Singapore; ^2^ Department of Chemical and Biomolecular Engineering 4 Engineering Drive 4 Singapore 117585 Singapore; ^3^ Institute of Materials Research and Engineering A*STAR (Agency for Science, Technology and Research) 3 Research Link Singapore 117602 Singapore

**Keywords:** atomic‐molecular design, energy applications, environmental applications, metal–organic frameworks, reticular chemistry

## Abstract

Continuing population growth and accelerated fossil‐fuel consumption with recent technological advancements have engendered energy and environmental concerns, urging researchers to develop advanced functional materials to overcome the associated problems. Metal–organic frameworks (MOFs) have emerged as frontier materials due to their unique porous organic–inorganic hybrid periodic assembly and exceptional diversity in structural properties and chemical functionalities. In particular, the modular nature and modularity‐dependent activity of MOFs and MOF derivatives have accentuated the delicate atomic‐ and molecular design and synthesis of MOFs, and their meticulous conversion into carbons and transition‐metal‐based materials. Synthetic control over framework architecture, content, and reactivity has led to unprecedented merits relevant to various energy and environmental applications. Herein, an overview of the atomic‐ and molecular‐design strategies of MOFs to realize application‐targeted properties is provided. Recent progress on the development of MOFs and MOF derivatives based on these strategies, along with their performance, is summarized with a special emphasis on design–structure and functionality–activity relationships. Next, the respective energy‐ and environmental‐related applications of catalysis and energy storage, as well as gas storage‐separation and water harvesting with close association to the energy–water–environment nexus are highlighted. Last, perspectives on current challenges and recommendations for further development of MOF‐based materials are also discussed.

## Graphical abstract

**Metal–organic frameworks (MOFs) are a preeminent class of nanoporous crystalline materials** regarding their modular nature that guides the preparation of chemically and structurally diverse frameworks, and renders them promising candidates for many potential applications. Synthetic controllability of MOFs and MOF derivatives toward targeted structural properties and chemical functionalities for energy storage/conversion, gas storage/separation, and water remediation/harvesting applications are discussed to unveil design–structure and functionality–activity relationships.

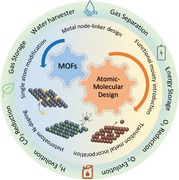

## 1 Introduction

Metal–organic frameworks (MOFs), also known as porous coordination polymers (PCPs) or porous coordination networks (PCNs), have become one of the most extensively investigated classes of materials in the fields of material science and chemistry, as revealed by the dramatically increasing number of MOF entities in Cambridge Structural Database (CSD) from 20 000 in 2013 to over 88 000 in 2018.^1^ Distinct from other traditional porous materials (e.g., zeolites and ordered mesoporous silica), the salient features of MOFs emanate from their modular nature and synthetic control toward targeted structural properties and chemical functionalities.^2^ Great diversity of metallic nodes and organic linkers, as molecular building blocks (MBBs), with gained knowledge on kinetics and thermodynamics of MOF crystal growth have paved the way for judicious selection of building blocks and predetermined coordination reaction under favorable conditions. This has directed the rational design and synthesis of numerous extended periodic networks and guided the understanding for structure–property–performance relationship.[qv: 2a,3] Unprecedented Brunauer–Emmett–Teller (BET) surface areas (extending beyond 10 000 m^2^ g^−1^) and porosities (up to 3.60 cm^3^ g^−1^) with tailorable and well‐defined dimensions, geometries and polarities have been the foremost features of MOFs.^4^

**Figure 1:**
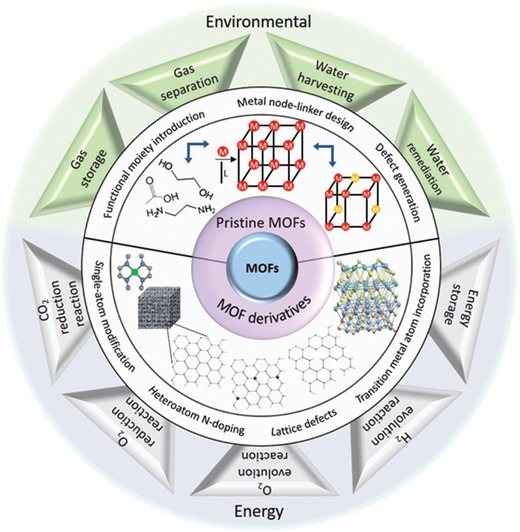
Schematic illustration for the atomic‐ and molecular‐level design strategies of MOFs and MOF derivatives along with the potential energy and environmental applications.

Sophisticated periodic structures with diverse topological and functional merits unveiled by exquisite control over reticular chemistry have provided impetus for MOF research toward design of materials for specific applications in both energy and environmental sectors, including gas storage,^5^ separation,[qv: 5b,d,6] water harvesting and remediation,^7^ and energy storage and conversion.^8^ The realization of these applications is closely reliant on the chemical functionality of metallic nodes and organic linkers within the MOFs, which is introduced either prior to/during de novo synthesis or through postsynthetic MBBs modification (PSM) subsequent to MOF formation, both relying on the atomic‐ and molecular‐level design.^9^ Meticulous assembly of organic linkers, linker functionalities, and metallic nodes prior to/during de novo synthesis can yield MOFs with desired chemical and physical properties such as steric and electronic environment, host–guest interactions, and pore structures that are of crucial importance in determining their potential in energy and environmental applications. Likewise, PSM strategies, such as MBBs modification and replacement, can provide additional degrees of freedom to modularity when the direct assembly of MBBs is hampered by limitations, including partial solubility of MBBs, undesired side reactions, and chemical and thermal instability.^9^ Specifically, inherent and intentionally created controllable defects (e.g., missing linker, missing node defects) engendered via de novo synthesis and PSM greatly influence the pore structures for selective gas adsorption and separation, hydrophilicity for water harvesting and electronic environment of catalytic active sites.[qv: 7c,10] Therefore, in depth understanding of the strategies for application‐based rational atomic‐ and molecular‐level engineering of pristine MOF chemistry is essential (**Figure** **1**).

Apart from the unique application‐targeted tunable properties of pristine MOFs leveraged by the modular chemistry and structure, transformation of the predesigned MOFs expands the materials choice to open up new opportunities. Because of the high carbon contents and well‐ordered open‐frameworks, MOFs are prompted to yield carbon‐based materials with high surface areas and porosities via controlled high‐temperature posttreatment reactions. Besides the inherent structural properties, carbon materials with distinct electronic properties are also desirable for energy applications. In this regard, demand for highly conductive carbon frameworks that can accommodate abundant active sites for electrochemical reactions to take place with optimum activation energy has facilitated the research endeavors on MOF‐derived carbons.^11^ Particularly, different from the perfect (defect‐free) carbon lattice, defective carbon analogues have been an important research hotspot since they have been reported to be electrochemically more active as a result of the surface charge redistribution and tailored reaction intermediate binding energies.[qv: 11g,12] Notably, defective carbons can be transformed from MOFs over reticular chemistry modulation and/or posttreatment condition tuning due to the formation of lattice disruption/reconstruction induced by the presence and/or removal of inherent non‐metal heteroatoms (e.g., N atoms) and metal nodes (e.g., metals with low boiling points). Moreover, deliberate control on reticular chemistry and posttreatment conditions plays a pivotal role in comprehending a structure–performance relationship over defects to pave the way for designing high‐performance materials. Atomic‐ and molecular‐level design strategies are capable of unveiling point and extended defects in MOF‐derived carbons such as N‐doping, lattice defects, and single atomic metal sites (Figure 1). In addition to carbons derived from MOFs, transition metal‐based materials (e.g., oxides, chalcogenides, pnictides, and nitrides) are widely prepared from MOFs on account of thermal and chemical susceptibility of the MOFs' coordination chemistry.[qv: 11e–g] Importantly, incorporation of alien heteroatoms to the transition metal derivatives lattice (Figure 1) endows the structure with enhanced electrical conductivity, electron density, and surface adsorption/desorption energy, which are vital in electrochemical energy storage and conversion processes. Thus, it is important to discuss the atomic‐ and molecular‐level engineering strategies to attain defective carbon and heteroatoms incorporated transition metal derivatives lattices from MOFs (Figure 1).

Here, we provide a broad yet focused review of the atomic‐ and molecular‐level strategies to design MOFs and MOFs derivatives for energy and environmental applications. First, we discuss reticular synthesis of MOFs and strategies to alter MOFs' structural properties and chemical functionalities. Afterward, approaches for modulation of pristine MOFs toward design of functional carbon materials and transition metal derivatives, and effects of postsynthetic modification conditions are summarized. In particular, MOF‐derived carbons with point and extended defects, such as N‐doping, lattice defects and single atomic metal sites, and heteroatom‐doped transition metal derivatives are emphasized. Next, their environmental and energy‐based applications, including gas storage, separation, water harvesting, energy storage and conversion, are discussed through recent case studies with a focus on performance‐targeted design. Finally, a summary is provided together with a critical discussion on challenges and future opportunities for atomic‐ and molecular‐level design of MOFs for energy and environmental applications.

## 2 Reticular Chemistry of MOFs

### 2.1 Selection of Metal‐Node Linker

As the primary constituents of the porous framework, the identity of the metal‐node and linker foreseeably holds wide‐ranging implications on the final synthetic outcome. As the first level of design, the geometric information encoded by the metal‐node based MBBs may be used to direct the connectivity and porosity of the MOF scaffold to a large extent. Isoreticular chemistry, which considers the design of crystalline materials based on the geometric contribution of MBBs, has enabled tailoring of new materials.[qv: 6b] Transition‐metal carboxylate clusters with defined polyhedral shapes,^13^ as well as infinite rod‐based building blocks may be employed toward the design of MOFs.^14^ Particularly, specific building blocks have exhibited exceptional tolerance toward metal substitution, with prominent examples such as the rod‐based MBB in MOF‐74 (M = Zn, Co, Ni, Mg, Fe, etc.)^15^ and hexanuclear MBB for high‐valence metals (M = Zr, Hf, Ce, Th, Ln, etc.).^16^ The robust formation conditions of the abovementioned clusters allow the properties of a framework to be varied, both subtly and dramatically. For gas storage applications, a lower atomic weight of the metal may be desired to enhance gravimetric gas uptake capacities. For instance, the magnesium analogue of MOF‐74 exhibits promising uptakes for CO_2_ and small hydrocarbons by virtue of its low atomic weight and other factors.[qv: 15c] In addition, the incorporation of certain elements may endow the framework with distinct material properties belonging to the metal, such as fluorescence in characteristic wavelengths (e.g., lanthanides, in particular Eu and Tb).^17^

The diversity of metals incorporated in the abovementioned frameworks belies the synthetic challenges, which may surface in attempts to generate isoreticular MOFs based on different elements. For example, while the prototypical hexanuclear zirconium cluster was employed for MOF synthesis by simple combination of metal salt and ligand, the rare‐earth based counterparts may only be generated in the presence of fluorinated linkers or structure‐directing agents.[qv: 16e,18] The additives are required presumably to stabilize the hexanuclear MBB and avoid hydrolytic polymerization of the metal precursor into multinuclear hydroxo‐based structures.[qv: 14a] Trigonal prismatic carboxylate clusters have been synthetically isolated across numerous elements as embodied by the mesoporous zeotype framework MIL‐100 (M = Al, Fe, Cr, V, Ti, etc.), which is constructed using the trigonal planar trimesate ligand.^19^ When similar metal precursors are combined with linear ditopic ligands such as terephthalic acid, isomorphs with different arrangements (e.g., MIL‐88) or MBBs (e.g., MIL‐53) may be generated, which significantly complicates optimization efforts.^20^ The preference for a certain coordination environment (i.e., octahedral vs tetrahedral) is another factor, which deserves consideration in the selection of metal node‐linker combinations. Structures involving tetrahedral coordinated M^2+^ cations like MOF‐5 have been facilely obtained using Zn precursors but not with other divalent transition metals such as Ni. Although doping of other divalent metals has been realized by solution exchange, the long time to equilibrium and low rate of incorporation suggest that such approaches are challenging to operate at scales relevant to practical applications.^21^

### 2.2 Incorporation of Functional Groups

Given the inexpensive nature of most metal precursors, the variation of metal nodes is an attractive way to realize compositional and structural diversity in MOFs. In parallel, linker selection provides additional degrees of freedom to direct the assembly of a desired scaffold structure. One of the earliest demonstrations of the isoreticular concept was to extend linear ditopic ligands by rigid phenylene units, dramatically augmenting the porosity of the self‐assembled materials.^22^ This technique of pore size expansion is now ubiquitous in the literature, being applied to other different MBBs (e.g., Zr_6_ of NU‐1000 series).^23^ For multitopic ligands, the linker extension is accompanied by changes in other geometric parameters such as the dihedral angles, which may further influence topological outcomes.^24^ Notably, the combination of hexanuclear MBBs with tetratopic ligands may result in an excess of 10 unique nets depending on node connectivity and various conformational properties.^25^ Where such parameters may be systematically controlled, the practice of isoreticular chemistry allows further opportunities to tune material properties such as the defects concentration, as seen in a recent example illustrating the use of transversal design in Zr‐MOFs by Guillerm et al.^26^ Nevertheless, the synthetic effort involved in precise manipulation of these parameters may be significant, yet conflict with other attempts to introduce nonstructural functional moieties.

Beyond the scaffolding role of the linker, the incorporation of functional moieties is a straightforward way to generate compositional diversity in MOFs. In linkers comprising phenylene units, aromatic substitution presents an avenue to a wide range of derivatives, including amino, nitro, halide, and sulfo‐functionalized compounds. Notably, the functionality on the aromatic rings has been used to systematically vary the electronic environments in the vicinity of the metal nodes, which, for example, significantly impacted the performance in photocatalysis for MIL‐125(Ti).^27^ Besides organic substituents, a major branch of research concerning the incorporation of metal‐chelating sites into MOFs, which mimics the highly efficient molecular catalysts found in nature has been demonstrated. 2,2′‐Bipyridine and porphyrinic motifs have been successfully incorporated in numerous MOF systems, whereas salen‐based ligands were also explored, with varying degrees of success.^28^ The potential participation of incorporated functional groups in the self‐assembly is undesirable and poses challenges to successful synthesis. Hard soft acid base theory has been employed to rationally optimize the synthetic process, by utilizing preferential coordinative tendencies to thermodynamically favor the desired product.^29^ However, it is likely that drastic changes in the synthetic conditions are necessary to compensate for interfering chelating effects by the introduced functional groups. As a recent example, a first synthesis of amino‐acid‐containing Zr‐MOFs has been reported in 2018 by Serre's group. While the linker (L‐aspartic acid) is substantially similar to a previously isolated analogue based on fumaric acid, synthesis under exceptionally concentrated solutions is necessary to compensate the competitive complexation of the Zr precursor by the zwitterionic linkers.^30^ Considering the challenges in direct synthesis, solid‐state transformations have been relied on as a simple, albeit more resource‐consuming, route to decouple the scaffold formation and the incorporation processes of functional groups.^31^

As a further elaboration of the concept of distinguishing structural and functional linkers, the chelation of nonstructural ligands to metal nodes has been employed to confer various desired properties. The term “solvent‐assisted ligand incorporation” (SALI) has been suggested by Farha et al. to describe this technique.^32^ By avoiding the self‐assembly process, SALI has a greater tolerance to functional groups. For instance, linear diamines have been introduced into the unsaturated metal centers, which facilitated a chemisorption mechanism for CO_2_ sequestration.^33^ Defects at the metal nodes of Zr‐MOFs have been shown to accept a wide range of chelating ligands, including monodentate carboxylates, sulfates, and phosphonates.^34^ The choice of chelating moiety is pertinent to ensuring the effective lifespan of the SALI functionalization against replacement by competing ligands. SALI is contingent on the presence of unsaturated metal sites in the framework, the formation of which will be discussed in a later section. In a parallel development, Zhou's group and others have reported a number of MOF platforms with appropriately sized pockets, which allow postsynthetic insertion of linkers with different functionalities.^35^ The base platforms are generated by using a scaffolding linker to favor highly defective structures; while the functionalized linkers may be introduced stepwise into their thermodynamically favored positions. Such a high level of spatial control is distinct from other one‐pot methods to generate mixed‐ligand frameworks with close to stochastic distributions.^36^

While the inorganic component of the MOF is typically concentrated at the metal nodes, some linkers may also exhibit substantially inorganic character. In particular, a diverse category of pcu MOFs is constructed by extension of octahedral pyridine‐based molecular building blocks, which are further crosslinked into 3D frameworks by fluorine‐containing anionic pillars.^37^ Apart from the linear silicon hexafluoride anion adopted in the prototypical SIFSIX‐1‐Zn pcu MOF, the identity of the “pillar metal” has been extended to various others, including Ti and Sn.^38^ Crucially, the replacement of the “pillar metal” permits the adjustment of porosity by exceptionally small increments (0.2–1 Å) within the ultramicroporous pore system, which has enabled benchmark performance among the existing materials for numerous demanded separations.^39^ The potentially low cost to implement these changes and minimal impact on the MOF scaffold poises inorganic “fine‐tuning” as a useful complement to organic modification for the atomic‐level design of functional MOF materials.

### 2.3 Introduction of Defects

The presence of unsaturated metal centers (UMCs) in MOFs has inspired numerous potential uses as reactive sites for the sorptive interaction with guest molecules, as catalytic active sites, or as grafting points for framework‐modifying atoms or ligands. Beyond the transient formation of UMCs as a result of their chemical environment, periodically distributed UMCs have been recognized in several MOF structures, such as Zn_3_(BDC)_3_·6CH_3_OH, the MOF‐74 series, and MIL‐101.[qv: 15a,40] Their presence, being highly synthetically reproducible and suitable for characterization by crystallographic methods, makes them highly relevant from the perspective of MOF designers. Dietzel et al. have rationalized certain contributing factors to UMC generation in MOF‐74 (CPO‐27), which include tolerance of metal nodes for changes in coordination number, high framework rigidity, as well as availability of compensating ligands.[qv: 15a,41]

**Figure 2:**
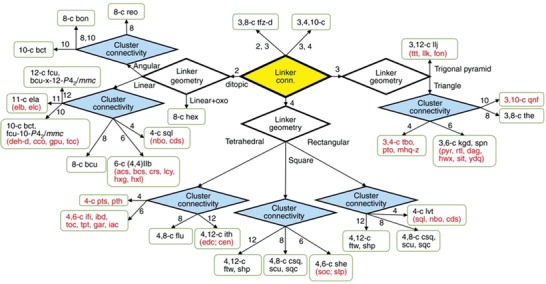
Topologies of Zr‐MOFs built on Zr_6_ clusters with ditopic (145) and polytopic (56) linkers. Reproduced under the terms of the CC‐BY Creative Commons Attribution 4.0 International License (http://creativecommons.org/licenses/by/4.0/).^25^ Copyright 2018, The Authors, published by Springer Nature.

More recently, Zr and Hf frameworks have gained the focus in MOF defect engineering owing to the convergence of the MOF properties to the abovementioned criteria. The hexanuclear Zr_6_ nodes have been reported to tolerate replacement of 6 (in the case of MOF‐808) and 8 (in the case of NU‐1400) coordination sites by nonstructural ligands in the presence of monocarboxylate “modulators” such as formic acid or acetic acid.^42^ Appreciation of variable connectivity in the metal nodes due to defects greatly facilitates efforts in topological prediction. Where a high concentration of defects is desired, linkers may be rationally selected to target “defective” nets with lower than ideal connectivity (**Figure** **2**). Introduction of geometric deviations by incorporation of ring heteroatoms or appending sterically bulky moieties are the key strategies to facilitate their synthetic isolation.^43^

## 3 Transformation of Designed MOFs into Functional Materials

### 3.1 MOF‐Derived Carbons

#### 3.1.1 Local and Extended Defects Generation

*Heteroatom N‐Doping*: The sp^2^‐hybridized carbon‐based materials possess electrochemically inactive π electrons, zero spin density, and uniform small charge density, which impart near electroneutrality to the surface and lead to inferior electrochemical activity in charge transfer‐dominated electrochemical reactions. Impressively, over the last decade, sp^2^‐hybridized carbon electronic structures have been effectively modulated by breaking the local electroneutral atomic bonding via single or multiple heteroatom doping using N, B, P, and S atoms, which are perceived as point defects.^44^ In particular, substitution of carbon atoms with nitrogen heteroatoms dramatically changes surface polarity of the neighboring carbon atoms in the framework due to large electronegativity difference between carbon (χ = 2.55) and nitrogen (χ = 3.04), which induces electron‐deficient carbon environment favorable for reactant adsorption. N atoms can be incorporated in various binding configurations in form of pyridinic‐N, pyrrolic‐N, graphitic‐N (or quaternary‐N), pyridinic oxide‐N, and pyridinium‐N.[qv: 44e,45] Doping content, chemical states and configurations of the nitrogen atoms are important factors determining the final activity of the carbon structure. For example, it was revealed that pyridinic‐ and graphitic‐N sites take active role in oxygen reduction reaction by reducing oxygen adsorption energy barrier, facilitating the electron transfer and weakening the O—O bond.^46^ Chen and co‐workers reported that graphitic‐N sites with low doping concentration are optimal sites for oxygen evolution reaction by analyzing the free energy‐change diagrams and electrostatic potential surfaces using density functional theory (DFT) calculations.[qv: 44e] Nevertheless, in supercapacitor applications, graphitic‐N enhances the electrical conductivity of the carbon framework, while pyridinic‐ and pyrrolic‐N play main role in improving pseudocapacitance due to their pseudocapacitive contribution in redox reactions.^47^

**Figure 3:**
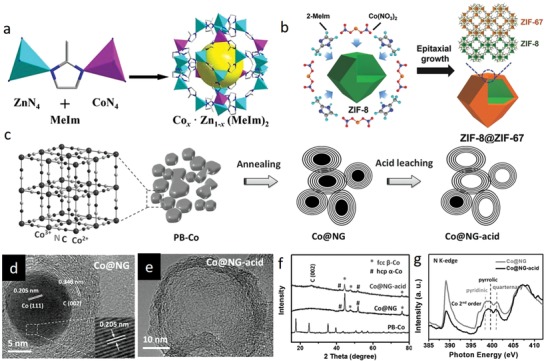
a) Schematic illustration of Zn/Co BMOF preparation via coprecipitation. Reproduced under the terms of the CC‐BY Creative Commons Attribution 4.0 International License (http://creativecommons.org/licenses/by/4.0/).^135^ Copyright 2016, The Authors, published by Springer Nature. b) Schematic illustration of Zn/Co BMOF preparation via coepitaxial growth. Reproduced with permission.[qv: 48o] Copyright 2017, Wiley‐VCH. c) Schematic illustration for preparation of nitrogen‐enriched graphene shells from cobalt‐containing Prussian blue (PB‐Co). TEM images of d) Co@NG and e) Co@NG‐acid. f) XRD patterns and g) N K‐edge XANES spectra of Co@NG and Co@NG‐acid. c–g) Reproduced with permission.[qv: 51c] Copyright 2016, Wiley‐VCH.

In this respect, MOFs are excellent precursors for preparation of porous heteroatom‐doped nanocarbon materials owing to their intrinsic properties such as high porosities, exceptional specific surface areas, tunable ligand‐metal sites, inherent N atoms, rich carbon contents, and favorable dynamic frameworks. As a subclass of MOFs, ZIFs are particularly advantageous organometallic complexes in view of their nitrogen‐ and carbon‐rich imidazolate (and its derivative) linkers and transition metal ions (e.g., Zn, Co, Ni, Cu, Fe), which could act as seeds to catalyze formation of highly graphitic carbons. To date, the most widely investigated ZIF candidates have been ZIF‐8 and ZIF‐67 for preparation of N‐doped carbons, where graphitization degree, nitrogen‐dopant content, dopant configuration, surface area and morphology of the final carbon structure have been examined and modified by engineering the parent ZIF precursors and tuning the posttreatment conditions.^48^ Upon direct carbonization of ZIF‐8 at high temperatures (>600 °C) under inert environment, ZIF‐8 tends to transform into microporous carbon framework with high degree of N doping and high surface area. Nonetheless, carbon is mostly amorphous and structural collapse occurs above 900 °C due to weak catalytic graphitization ability and evaporation of Zn metallic nodes (mp 420 °C, bp 907 °C).[qv: 48b,e,i,l,49] By contrast, ZIF‐67 crystals, isostructural to ZIF‐8, can be converted into a well‐graphitized carbon structure by direct thermolysis since Co nanocrystals, reduced from Co^2+^ nodes during thermolysis, catalytically assist formation of graphitic layers. However, unlike the ZIF‐8‐derived carbons, surface area and N content are sacrificed in ZIF‐67‐derived carbons. Individual benefits of these isostructural ZIFs‐derived carbons could be merged by rational design of the metallic nodes without compromising the morphological merits by leveraging similar ionic radii and coordination number of Zn and Co ions as well as similar unit cell parameters (α_ZIF‐8_ = 16.99 Å, α_ZIF‐67_ = 16.95 Å). Such a bimetallic organic framework (BMOF) design is attainable via coprecipitation (**Figure** **3**a) or epitaxial growth (Figure 3b) methods. To date, BMOFs coprecipitation method has been widely investigated by precisely tuning Co and Zn molar ratios to study the porosity, surface area, graphitization degree, and N‐doping content/configuration of the derived carbons.[qv: 48j,l,n] It has been reported that the optimum zinc incorporation into Co‐ZIF framework and its direct carbonization at 700–1000 °C under inert atmosphere compensates minimized the surface area, microporosity, and N‐content of ZIF‐67‐derived graphitic carbons. Zinc species regulate the graphitization kinetics of Co by precluding excessive growth and aggregation of catalytically active Co nanoparticles, thereby leads to gradual transition from amorphous to graphitic carbon. At optimum Zn/Co molar ratios, BMOF‐derived carbons exhibit Co nanoparticles (within the size range of 9–11 nm) wrapped in circular well‐graphitized carbon layers possessing moderate nitrogen content (<9.0 wt%) in form of graphitic‐, pyrrolic‐, and pyridinic‐N. Importantly, it has been demonstrated that tuning the Zn/Co molar ratio affects N‐doping configurations in the final carbon product.[qv: 48j] Alternatively, by the epitaxial growth method, nanoporous N‐doped carbon@graphitic carbon (NC@GC) structures with high surface areas and N contents have been also attained by synthesis of ZIF‐8@ZIF‐67 crystal and its successive thermolysis.[qv: 48b,o,50]

Apart from ZIF‐based MOF_S_, other nitrogen‐containing MOFs can be employed to prepare N‐doped carbonaceous materials.^51^ Jeon et al. obtained nitrogen‐doped porous carbons from the pyrolysis of a nitrogen‐containing isoreticular metal–organic framework (IRMOF‐3, containing Zn and 2‐aminoterephthalic acid) at various temperatures (600 °C, 700 °C, 800 °C, and 950 °C).[qv: 51a] Pyrolysis is capable of altering the nitrogen content, graphitization degree, disorder/defects and porosity, indicating the significance of posttreatment conditions on atomic structure of the as‐prepared samples. The decomposition of the 2‐aminoterephthalic acid ligand in IRMOF endowed the final carbon structure with N atoms, in which the N loading decreased from 7 to 3.3 wt% as the temperature increased from 600 °C to 950 °C. Another commonly utilized N‐containing MOFs are Prussian blue analogues (PBAs), designated as *A_x_*M′[M″(CN)_6_]*_y_*. ⊙ _1‐_ *_y_*⋅ *n*H_2_O, where M′and M′′ are transition metals coordinated with carbon and nitrogen.[qv: 51b–d,52] Taking cobalt analog of Prussian blue for example, Zeng et al. prepared nitrogen‐enriched graphene shells via pyrolysis of Co_3_[Co(CN)_6_]_2_ (Figure 3c).[qv: 51c] Notably, abundant Co NPs were formed through the aggregation of Co nodes at elevated temperatures (above 600 °C) due to high Co/C ratio in PBA compared to ZIFs. Because of the richness and strong catalyzing effect of Co NPs, thick N‐doped graphene shells (5–16 layers) evolved around the Co NPs. The Co NPs (≈20 nm) cores interlaminated in the thick graphene shells (Co@NG) (Figure 3d) were then leached out via acid treatment to fully realize the N‐rich graphene shells (Co@NG‐acid) (Figure 3e) as also confirmed by X‐ray diffraction (XRD) and X‐ray absorption near‐edge structure (XANES) studies (Figure 3f,g). This study revealed that the concentration of metallic node relative to carbon is a crucial factor affecting the synergistic interaction of the inherent MOF components during pyrolysis, which determines the final graphitic morphology.

For the MOFs precursors with absence of N in the organic ligand, such as Zn‐MOF‐74, MIL‐101 (Fe), MIL‐100 (Fe), MIL‐88B, MOF‐5, and NENU‐5, N atoms still can be extrinsically incorporated into the MOF‐derived carbons via the thermal treatment of the MOFs with external N sources (e.g., melamine, dicyanamide, poly(cyclotriphosphazene‐co‐4,40‐sulfonyldiphenol, ammonia, triethylamine, urea, etc.).^53^

**Figure 4:**
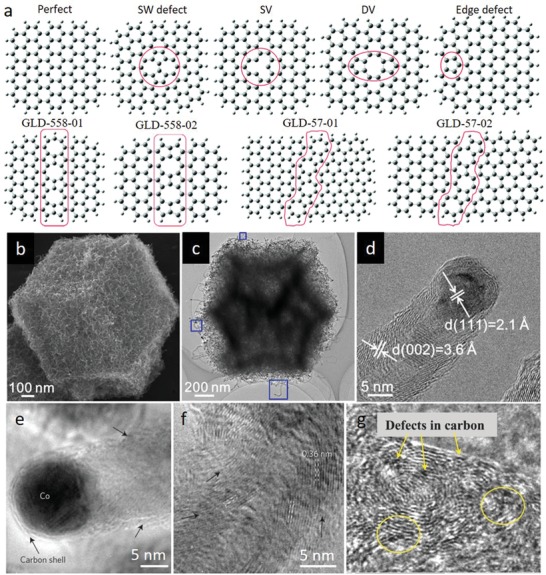
a) Perfect and defective graphene clusters. Reproduced with permission.[qv: 12a] Copyright 2015, The Royal Society of Chemistry. b) SEM and c) TEM images of the N‐doped CNTs framework derived from ZIF‐67. d) HRTEM image of a typical defective and edge‐rich CNT with encapsulated Co nanoparticle. b–d) Reproduced with permission.[qv: 48e] Copyright 2017, American Chemical Society. e,f) HRTEM images of the N‐doped CNT with divergent graphitic layers. Arrows in (e) and (f) indicate the direction of the graphitic layers. e,f) Reproduced with permission.[qv: 48g] Copyright 2016, Springer Nature. g) HRTEM image of Fe‐SNC showing the defective graphene nanosheets. Reproduced with permission.^55^ Copyright 2016, Wiley‐VCH.

*Lattice Defects*: Apart from the inherently existing heteroatom dopants as point defects, MOF‐derived carbon lattice can intrinsically accommodate other types of extended and point defects (e.g., broken fringes, holes, dislocations, grain boundaries, Stone–Wales (SW) defects and single vacancy (SV)/double vacancy (DV), etc. (**Figure** **4**a)) during high‐temperature transformation of MOF precursors due to formation of lattice disruption/reconstruction induced by presence (e.g., N atoms) and/or removal of inherent heteroatoms (e.g., N atoms) and metal nodes (e.g., metals with low boiling points).[qv: 12a,48e,g,54] Recently, Meng et al. reported a facile strategy for oriented formation of CNTs from MOFs (e.g., ZIF‐67, Co, Fe‐ZIF microspheres, Ni‐ZIF microspheres, Co‐BTC microsheets, Ni‐BTC hollow microspheres, Co‐MOF nanorods, and ZIF‐67@ZIF‐8) through a low temperature (435 °C) direct pyrolysis process under argon.[qv: 48e] When the pyrolysis temperatures are held close to decomposition temperature of the MOFs, small Co, Ni, and Fe nanocatalysts gradually formed from metallic nodes and served as seeds to initiate the subsequent transformation of organic units into CNTs. Eventually, N‐rich CNTs (up to 25.5 wt%), obtained from N‐containing zeolitic‐imidazole type parent MOFs, exhibited randomly oriented and stacked graphitic layers with abundant defects and edges (Figure 4b–d). Nonetheless, successive and integrated graphitic layers with low defect density formed in pure CNTs, which were derived from other MOFs with N‐deficient linkers (Ni‐BTC, Co‐BTC, and Co‐MOF), signifying the important role of N‐heteroatoms in generating structural extended defects in graphitic carbon lattice. Similarly, it was demonstrated that N‐doping and defects in CNTs synchronously emerge during high temperature (>600 °C) pyrolysis of N‐containing ZIF‐67 in presence of Ar/H_2_ atmosphere as revealed by transmission electron microscopy (TEM) and high‐resolution TEM (HRTEM) images (Figure 4e,f).[qv: 48g] Defect density and nitrogen doping were reported to decrease with increasing temperatures due to instability of N atoms and increase in graphitization degree. Naveen et al. further observed defective lattice on N and S co‐doped carbon, obtained from heteroatom‐rich polymeric zinc organic framework templates (ZnDTO).^55^ As shown in Figure 4g, N and S co‐doped porous carbon materials (Fe‐SNC) with well‐defined interconnected graphene nanosheets with abundant defects were obtained via direct carbonization of ZnDTO and hemoglobin (Fe source).

Aside from the extended defects induced by the existence of heteroatoms, vacancy‐based intrinsic defects can be created in MOF‐derived carbon materials by selectively removing metal and/or nitrogen heteroatoms from the carbon framework during pyrolysis process.[qv: 54b,g] Combining experimental data with theoretical calculations, it was reported that defect‐rich pure carbons could exhibit better activity than defect‐free heteroatom‐doped carbons.[qv: 12a,b,54b,g] Yao's group demonstrated that removal of N atoms from carbons could create G585 defect on graphene, which exhibits comparable activity to Pt in all steps for the ORR. It was reported that single nitrogen atom vacancy in N‐doped carbons causes instability in the lattice and combines with another vacancy to generate a thermodynamically stable nonhexagonal rings defect, referred as G585 defect.[qv: 54g] Along with the theoretical calculations, experimental studies were carried out to eliminate nitrogen atoms in the porous organic framework‐derived carbons through pyrolysis, where the removal of N leads to increased *I*_D_/*I*_G_ ratios. More recently, the same research group investigated formation of vacancy‐defects in pure‐carbon lattice by removing Zn atoms from IRMOF‐derived carbon (PC‐I8‐950).[qv: 54b] N‐doping effect was eliminated by selecting a nitrogen‐lack linker containing IRMOF, and Zn atoms were completely removed with flowing N_2_ at 950 °C. It was proposed that the removal of metal from sp^2^ carbon breaks the integrity and creates defects/disorders in PC‐I8‐950, which was reported to decrease the overpotential requirement for ORR.

#### 3.1.2 Single‐Atom Based Modification

Besides the transformation of intrinsic MOFs metallic nodes to metal nanoparticles for designing well‐graphitized defective carbon frameworks, the same metal nodes can be converted into atomically dispersed metal species in carbon framework in form of single‐atom metal sites. Single‐atom metal sites can be defined as atomically dispersed metal species, which are enclosed in the lattice with other type of atomic species and isolated from short‐range same‐type metal–metal interactions. Downsizing the metal nanoparticles or clusters into single atoms is of essential importance in energy‐related applications, particularly in catalysis, as it offers utmost atomic utilization and augmented charge transfer interactions of the single atoms with local coordination environment owing to the lowest size limit and structural effects.^56^ Specifically, single‐atom metal sites are ideal structures for fundamental understanding of catalytic reactions at the molecular level, since they possess homogeneously dispersed catalytic active centers and coherent atomic interactions. However, advance in single‐atom catalysts is mostly hindered by: i) low single‐atom density (<1 wt%) and ii) high mobility and sintering tendency of single atoms due to their excess surface free energies.[qv: 56b,c,57] Weak support–metal interactions eventually cause metal nanoparticle aggregates, further affecting the final performance of the material. Thus, in the last decade, rational synthetic strategies have been introduced to increase atomic loading amount and enhance metal–support interactions to assure atomic dispersion of metallic species. Mass‐selected soft‐landing,^58^ atomic layer deposition (ALD),^59^ and wet‐chemistry routes[qv: 56b,60] have been recognized as the strategies for preparation of single‐atom metal sites. Mass‐selected soft landing technique enables controlled selection of metallic species by size via a quadrupole mass filter and deflector assembly, and uniform deposition onto a support (such as magnesium oxide, titanium oxide, alumina).^58^ Similarly, ALD technique allows deposition of atoms on substrates with precise size and distribution via successive surface reactions.^59^ However, despite the controllability and sensitivity of mass‐selected soft‐landing and ALD techniques, practical applicability of them is significantly hampered by high instrumentation cost and low product yield. On the other hand, a more feasible and low‐cost strategy for preparation of single‐atom sites involves wet‐chemistry routes, in which single‐atoms are anchored on designated sites of supports through strong metal–support chemical interactions. In wet‐chemistry routes (e.g., coprecipitation, deposition–precipitation, impregnation, and photochemical method), a prerequisite is to devise support materials wisely to unveil active sites for trapping and strong stabilization of single‐atom sites.[qv: 56b,60] Defects, such as vacancies or unsaturated sites, on supports play a pivotal role as active sites since they introduce unique the electronic and coordination environment for favorable attachment of single atoms. Recent studies elucidated that wet‐chemical routes could induce numerous anion (O^2−^, S^−2^)[qv: 60a–e] and cation (Ti^4+^, Fe^+2^, Al^3+^, Ce^4+^, Ni^2+^)[qv: 56b,60f–h] vacancies on substrates, which can effectively capture and strongly stabilize various single‐atoms, including noble and transition metal atoms (Pt, Au, Ir, Pd, Co, Fe).

MOFs have been widely regarded as promising template materials for preparation of single‐atoms thanks to their tunable metal node‐linker functionalities, high porosities, and reactive coordination sites. Compared to other wet‐chemistry routes, MOF‐directed strategy enables a top‐down design, which is attained by atomic dispersion of single metal species in MOFs via spatial confinement and/or coordination interaction, followed by stabilization with posttreatment. MOF‐directed strategy assists preparation of earth‐abundant 3d transition metal single atom sites as well as noble metal atom sites with high loading in carbonaceous supports, which differs from traditional wet‐chemical routes that generally results in noble metal atomic species dispersion in metal oxides supports with low single atom content. Isolated metal atoms in carbon materials considerably alters the electrocatalytic activity of pure carbons due to modified electronic properties of carbon and low‐coordination environment of single atoms. Single‐atom sites can be anchored in MOFs and stabilized using various strategies such as intrinsic ionic coordination, pore confinement, NPs‐to‐single atom conversion and post‐treatment with stabilizing N source (**Table** **1**).

**Table 1:** MOFs for generation of single‐atom sites

MOF	Single‐atom	Strategy	Support	Loading [wt%]	Application	Refs.
UiO‐66–NH_2_	W	Intrinsic ionic coordination	N‐doped carbon	1.2 (ICP‐OES)	Electrocatalytic HER	[qv: [63e]]
PCN‐222 (MOF‐545)	Fe	Intrinsic ionic coordination	N‐doped carbon	1.7 (ICP‐AES)	Electrocatalytic ORR	62
ZIF‐8	Fe	Intrinsic ionic coordination	N‐doped carbon	0.45 (XPS)	Electrocatalytic ORR	[qv: [61c]]
Zn/Co–BMOF	Co	Intrinsic ionic coordination	N‐doped carbon	4.3 (ICP‐OES)	Electrocatalytic ORR	[qv: [61a]]
Zn/Co–BMOF	Co	Intrinsic ionic coordination	N‐doped carbon	0.34 (XPS)	Electrocatalytic ORR	[qv: [61d]]
Zn/Co–BMOF	Co	Intrinsic ionic coordination	N‐doped carbon	1.70 (ICP‐MS)	Electrocatalytic ORR	[qv: [61e]]
Zn/Co–BMOF	Co	Intrinsic ionic coordination	N‐doped carbon	0.25 (ICP‐AES)	CO_2_ electroreduction	[qv: [61b]]
MOF‐525	Co Zn	Intrinsic ionic coordination	MOF‐525	6.0 6.4 (ICP‐OES)	CO_2_ photoreduction	[qv: [63b]]
Al porphyrinic MOF (Al‐TCCP)	Pt	Intrinsic ionic coordination	Al porphyrinic MOF (Al‐TCCP)	0.07 (ICP‐AES)	Photocatalytic H_2_ evolution	[qv: [63d]]
PCN‐222	Ir/Pt Ir Pt Ru Au Pd	Intrinsic ionic coordination	PCN‐222	1.0/2.5 1.4 2.7 1.9 1.1 3.6 (ICP‐OES)	Photocatalytic H_2_ evolution	[qv: [63a]]
UiO‐66–NH_2_	Ru	Intrinsic ionic coordination	N‐doped carbon	0.3	Selective hydrogenation of quinoline	[qv: [63c]]
Hf–MOF‐808	V	Intrinsic ionic coordination	Hf–MOF‐808	1.1 (ICP‐OES)	Selective alcohol oxidation	[qv: [65f]]
Zr–NU‐1000	V	Intrinsic ionic coordination	Zr–NU‐1000	1.0 (ICP‐OES)	Selective alcohol oxidation	[qv: [63f]]
ZIF‐8	Fe	Pore confinement	N‐doped carbon	2.1 (ICP‐OES)	Electrocatalytic ORR	[qv: [64a]]
ZIF‐8	Ni	Pore confinement	N‐doped carbon	1.5 (ICP‐AES)	CO_2_ electroreduction	[qv: [64b]]
Ni–MOF	Ni	NPs‐to‐single atom conversion‐electrochemical activation	N‐doped carbon	1.5 (ICP‐AES)	Electrocatalytic HER	[qv: [66a]]
ZIF‐8	Pt Pd Au	NPs‐to‐single atom conversion‐pyrolytic activation	N‐doped carbon	0.41 0.16 0.18 (ICP‐AES)	Semihydrogenation of acetylene	[qv: [66b]]
Cu–MOF	Cu	Posttreatment with N	N‐doped carbon	20.9 (ICP‐OES)	Electrocatalytic ORR	65
ZIF‐8	Zn	Nanostructuring	N‐doped carbon	11.3 (ICP‐AES)	Photothermal catalytic CO_2_ conversion	136
ZIF‐8	Mn	Two‐step doping/adsorption	N‐doped carbon	3.03 (ICP‐MS)	Electrocatalytic ORR	87
ZIF‐8	Cu Ni Co	Gas‐migration	N‐doped carbon	0.54 0.31 0.24 (ICP‐AES)	Electrocatalytic ORR	137
ZIF‐8	Fe	Gas‐migration	N‐doped carbon	1.0 (XPS)	Electrocatalytic ORR	86

**Figure 5:**
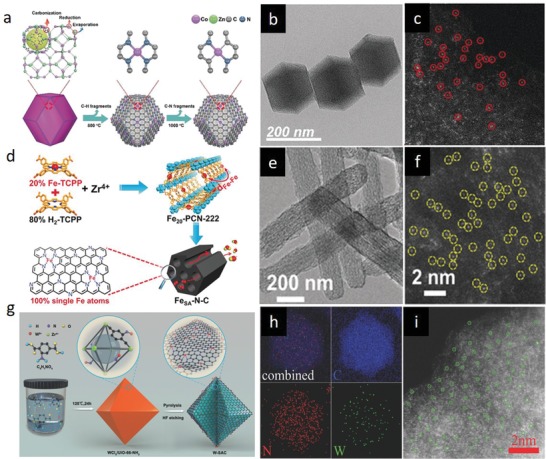
a) Schematic for the synthesis of single Co atoms in N‐doped carbon derived from Zn/Co BMOF. b) Scanning electron microscopy (SEM) image of the N‐doped carbon and c) HAADF‐STEM image showing the atomic dispersion of Co atoms. a–c) Reproduced with permission.[qv: 61b] Copyright 2018, Wiley‐VCH. d) Schematic for the synthesis of Fe_20_–PCN‐222 and single Fe atoms in N‐doped carbon (Fe_SA_–N–C). e) SEM image of Fe_SA_–N–C and f) HAADF‐STEM image showing the atomic dispersion of Fe atoms in Fe_SA_–N–C derived from Fe_20_–PCN‐222. d–f) Reproduced with permission.^62^ Copyright 2018, Wiley‐VCH. g) Schematic illustration for the synthesis of atomically dispersed W in N‐doped carbon (W‐SAC) from UiO‐66–NH_2_. h) Energy‐dispersive X‐ray (EDX) spectroscopy elemental mappings of the W‐SAC, indicating that W element is homogeneously distributed in W‐SAC. i) HAADF‐STEM image showing the atomic dispersion of W atoms. g–i) Reproduced with permission.[qv: 63e] Copyright 2018, Wiley‐VCH.

*Ionic Coordination*: In the intrinsic ionic coordination‐based single‐atom site creation, uncoordinated organic ligands and metallic nodes of MOFs play a crucial role in assembling metal ions within the 3D periodic coordination entities. In this strategy, single metal atom species are held in the framework with appreciable atomic separation to restrain further aggregation through the formation of metallic bonds. Meanwhile, N‐defects, formed during the postprocessing, act as coordinators to stabilize the single metal atoms. In this context, the most widely studied MOF has been the Zn/Co BMOF.^61^ In the pioneering work, Yin et al. synthesized a BMOF by tenaciously incorporating volatile Zn^2+^ nodes into the Co‐ZIF (ZIF‐67) framework.[qv: 61a] The inherent Zn^+2^ nodes in the Co‐ZIF were exploited to ensure spatial separation between Co^+2^ nodes to prevent them from aggregation during the high‐temperature posttreatment. Following the BMOF synthesis, Zn nodes were then evaporated at high temperatures over 800 °C, resulting in formation of N‐rich defects. Eventually, the uncoordinated N sites firmly trapped and stabilized the in situ reduced Co atoms as single atoms with a metal loading of over 4 wt%. It is worth mentioning that the ratio of Zn to Co directly influences single atom to NPs transition. Inspired by this study, the same strategy has been successfully implemented to regulate the coordination environment and the number of atomically dispersed Co catalysts considering the significant effect of the local coordination environment on activity and durability of single‐site catalysts (**Figure** **5**a–c).[qv: 61b] By altering the carbonization temperature, a series of atomically dispersed Co catalysts with N coordination numbers of 4, 3, and 2 were selectively obtained at 800 °C, 900 °C, and 1000 °C from Zn/Co BMOFs, respectively. Carbonization at higher temperatures led to formation of lower Co–N coordination numbers due to accelerated breakage and evaporation of C–N fragments with higher energy input. The atomically dispersed Co species were further evidenced by high‐angle annular dark‐field scanning transmission electron microscopy (HAADF‐STEM) and X‐ray absorption fine structure (XAFS) spectroscopy measurement.

Aside from employing mixed metallic nodes as in BMOFs, mixed ligands can be combined in MOFs to selectively attain homogeneous spatial separation between the potential single atoms. Recently, Jiao et al. reported preparation of high content (1.76 wt%) single Fe sites supported on N‐doped porous carbon through a mixed‐ligand strategy using a porphyrinic parent MOF, PCN‐222 (also called MOF‐545 or MMPF‐6).^62^ A series of Fe*_x_*–PCN‐222, including Fe_0_–PCN‐222, Fe_20_–PCN‐222, and Fe_40_–PCN‐222, were synthesized based on the assembly of H_2_‐TCCP (TCPP = tetrakis (4‐carboxyphenyl)porphyrin) and Fe‐TCCP ligands with Zr^4+^ metallic nodes, as depicted in Figure 5d. Among them, Fe_20_–PCN‐222 was successfully converted into single‐atom iron‐implanted N‐doped porous carbon (Fe_SA_–N–C) upon pyrolysis (Figure 5e,f), while Fe_40_–PCN‐222 was transformed into Fe NPs decorated N‐doped carbon due to agglomeration tendency of packed Fe ions with shortened Fe···Fe distance.

It should be noted that besides the insertion of potential single‐atom species as MOFs' inherent fragments during MOF synthesis, the same species could be trapped by the MOFs after synthesis through rich uncoordinated sites such as porphyrin centers in porphyrinic MOFs and —NH_2_ groups in UiO‐66–NH_2_.^63^ For example, using UiO‐66–NH_2_ as the precursor, Chen et al. fabricated N‐doped carbon matrix featuring well‐dispersed single W atoms (W‐SAC) (Figure 5g–i).[qv: 63e] The strategy is based on the strong coordination between WCl_5_ and uncoordinated amine groups (—NH_2_) in the MOF framework. The W‐anchored UiO‐66–NH_2_ polyhedrons were then decomposed into the target W‐SAC through a pyrolysis process at 950 °C. Importantly, without the assistance of the —NH_2_ groups, the W precursor was reported to aggregate and form 1.21 wt% W clusters or NPs, indicating the strong confinement and stabilization of single atoms via MOF functional groups.

*Pore Confinement*: Confinement of single‐atom metal precursors in periodic MOFs pores is another strategy toward design of single‐atom sites. Through a subsequent pyrolysis process, the resulting MOF can stabilize the pore‐confined single‐atom sites in carbon network to yield single‐atom carbons. Recently, Li's group reported single‐atom (Fe and Ni) decorated N‐doped porous carbon obtained from the facile pyrolysis of pore confined metal precursors in ZIF‐8.^64^ For example, Fe(acac)_3_ (molecular diameter, ≈9.7 Å), as the Fe single‐atom source, was trapped inside the ZIF‐8 pores (pore diameter, ≈11.6 Å) via a solution‐coprecipitation approach using zinc nitrate and 2‐methylimidazole, as building units, with Fe(acac)_3_.[qv: 64a] Then, thermal conversion of the MOF was performed at 900 °C under Ar atmosphere. During this process, migration and agglomeration tendency of Fe species is hindered by the uncoordinated N atoms, evolved by evaporation of Zn nodes, which leads to stabilization of isolated single iron atoms in N‐doped carbon framework with Fe loading up to 2.16 wt%. In a similar study, Ni single atoms were generated and distributed in nitrogen‐doped porous carbon by employing ZIF‐8 to confine Ni single‐atom source (Ni(NO_3_)_2_).[qv: 64b]

*Posttreatment with a Stabilizing N Source*: The coordinating and stabilizing effect of N is typically stimulated during the pyrolysis of N‐containing MOFs. Yet, concurrently, the inherent N sites in MOFs are gradually lost from the template at high temperatures due to CN decomposition, leading to a low N content in the as‐prepared single‐atom carbonaceous framework and limiting the single‐atom metal loading (<10 wt%). In this regard, posttreatment of MOF with external stabilizing nitrogen source emerges as an alternative route for enhancing the single‐atom sites content.^65^ As a proof of concept, by pyrolyzing a nitrogen‐free Cu–MOF (Cu(BTC)(H_2_O)_3_) with dicyandiamide (external N source), Li et al. prepared ultrathin nitrogenated carbon nanosheets hosting high amount of single Cu atoms (Cu–N–C) (20.9 wt%). During the process, abundant N atoms in dicyandiamide were reported to capture the Cu atoms in the MOF, establish a strong coordination and stabilize them in the carbon matrix. Control experiments revealed that only 200–300 nm sized Cu microspheres could be obtained in Cu–C without the protection and stabilization of the N atoms in dicyandiamide. Moreover, 1D structure of the parent MOF was transformed into 2D structure via the reaction of dicyandiamide with the carboxylic acid groups in the trimesic acid precursor, further improving the active surface area.

**Figure 6:**
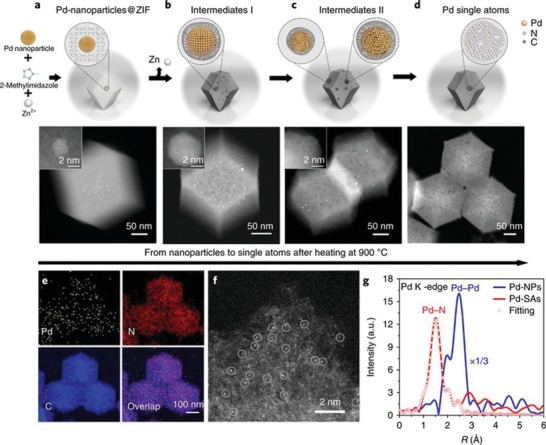
a–d) Schematic illustration for the Pd NPs to Pd single atoms (Pd‐SAs) transformation process along with the HAADF‐STEM and high‐resolution HAADF‐STEM (insets) images of the intermediates. e) EDX elemental mapping revealing the uniform distribution of Pd and N on the ZIF‐8‐derived carbon support. f) High‐resolution HAADF‐STEM image showing the atomic dispersion of Pd atoms. g) EXAFS spectra for Pd NPs and Pd‐SAs to confirm the atomic dispersion of Pd atoms. a–g) Reproduced with permission.[qv: 66b] Copyright 2018, Springer Nature.

*NPs‐to‐Single‐Atom Conversion*: Intrinsic ionic coordination and pore‐confinement strategy could endow carbon lattice with single‐atom sites depending on: i) coordination or confinement tendency of single‐atom sources to the parent MOF, ii) intrinsic stabilization capability of decomposed MOF during pyrolysis (via defect‐rich structure, e.g., uncoordinated N sites, vacancies), and iii) pyrolysis temperature. However, previous reports revealed that single‐atom to NPs transformation is very sensitive to the selection of MOF functional groups, linkers, and adjustment of pyrolysis temperature.[qv: 61a,63b,c,e] Thus, instead of impeding single atoms to NPs aggregation, reverse top‐down processes can be explored to break down the NPs into single‐atom sites.^66^ In this area of research, the pioneering work reported by Fan et al. revealed that electrochemical cyclic‐potential activates formation of isolated single Ni atoms from Ni NPs within graphitic carbon.[qv: 66a] Pyrolysis of Ni‐MOF at 700 °C in nitrogen (N_2_) atmosphere generated Ni NPs encapsulated in graphene layers (Ni@C). After removal of redundant Ni metal by HCl treatment, remaining small Ni NPs were atomized into single atoms by electrochemical constant‐potential and cyclic‐potential activation. Recently, Wei et al. demonstrated a controlled pyrolysis process to downsize various noble‐metal NPs (Pd, Pt and Au) embedded in ZIF‐8 into single atoms (**Figure** **6**a–d).[qv: 66b] They unveiled that thermodynamically stable coordination of Pd with N defects, which emerge during collapse of CN in ZIF‐8, favors atomization process over sintering at high temperatures. In the NPs to single‐atom site transformation process, ZIF‐8 decomposes during pyrolysis and generates N‐rich defects, which strongly capture the mobile metal atoms detached from NPs and prevents from Pd—Pd bond formation since atomization is dominant over sintering at high temperatures (≈900–1000 °C) due to sufficient energy input to overcome the kinetic barrier for single‐atom formation. Uniform Pd distribution was confirmed by EDX studies (Figure 6e), atomic dispersion of Pd atoms in the sample was directly observed by high‐resolution HAADF‐STEM (Figure 6f) and the coordination environment was further verified by extended x‐ray absorption fine structure (EXAFS) measurements (Figure 6g).

### 3.2 MOF‐Derived Transition Metal Oxides, Chalcogenides, Pnictides, and Nitrides

Labile coordination bonds between transition metal nodes (Cu, Co, Fe, Mn, Cr, Zn, etc.) and organic ligands make MOFs promising precursors for synthesis of a variety of transition metal derivatives, including metal oxides, chalcogenides, pnictides, and nitrides. The nanostructured MOF derivatives are typically obtained by calcining MOFs under reactive atmospheres (e.g., O_2_, NH_3_, NaH_2_PO_2,_ Se, S) or processing them with chemical reagents through solvothermal/hydrothermal reactions. During the transformation process, MOF decomposition is realized via homogeneous coordination bond breakage by a virtue of weak periodic interconnection of inorganic nodes with organic ligands, which subsequently gives rise to chemical interactions under feasible reactive conditions. In this respect, further understanding of the thermal and chemical susceptibility of the coordination chemistry allows rational transformation of the precursors into desired functional materials with tailored physicochemical properties such as bandgap, electrical conductivity, electron density, and surface adsorption/desorption energy. Particularly, nanoscale heterointerface generation and surface atomic arrangement through these transformation processes offer unprecedented electronic structures, which could be advantageous in electrochemical energy storage and conversion processes.

*Tranistion‐Metal Incorporation*: Incorporating additional transition metal atoms into the host MOFs not only introduces a synergistic effect due to cooperation of the different metal functionalities but also unveils heterointerfaces that offer exceptional electronic effects beneficial for mass and electron transfer, which is not attainable in the monometallic host MOF nanostructures. MOFs containing different type of metal centers can be synthesized via various strategies such as: i) substitution of parent metal nodes with metal ions possessing the same valency, ii) confinement of guest metal functionalities in the MOF cavities, and iii) ion‐exchange reactions.[qv: 15c,67] These facile synthetic modulations have inspired the design of multitopic MOFs for intrinsic transition metal incorporation to the MOF derivatives, offering great opportunities for rational functionalization of MOF derivatives.

**Figure 7:**
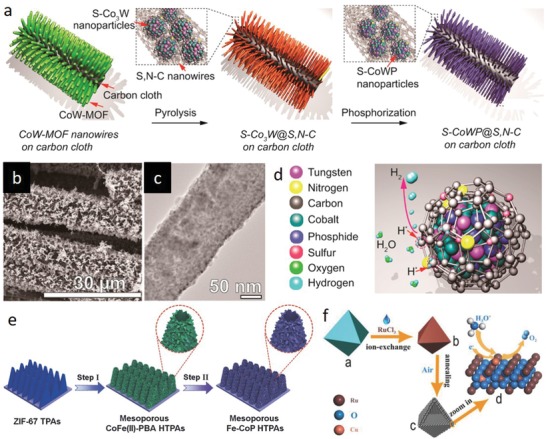
a) Schematic procedure for preparation of S‐CoWP@S,N–C nanowires on carbon cloth by two‐step pyrolysis‐phosphorization strategy. b) SEM and c) TEM images of the S‐CoWP@S,N‐C. d) Schematic of S‐ and N‐doped carbon‐wrapped CoWP nanoparticles. a–d) Reproduced with permission.[qv: 68a] Copyright 2018, American Chemical Society. e) Illustration for the synthesis of mesoporous Fe incorporated CoP hollow triangular plate arrays (Fe‐CoP HTPAs). Reproduced with permission.[qv: 68c] Copyright 2018, Wiley‐VCH. f) Illustration for the synthesis of Cu‐doped RuO_2_ hollow porous polyhedron. Reproduced with permission.[qv: 68b] Copyright 2018, Wiley‐VCH.

To date, mixed metal derivatives have been readily obtained through processing the transition metal doped MOFs due to existence of various metal species in the framework.^68^ For example, Weng et al. have demonstrated a MOF‐based strategy for incorporation of W into CoP by employing Hofmann‐type Co and W containing MOF nanowires (CoW‐MOF) (**Figure** **7**a–c).[qv: 68a] The parent MOF, synthesized via the solid–liquid reaction of Co‐MOF with (Bu_3_N)_3_W(CN)_8_, was reported to possess bipyridine (connecting adjacent Co atoms) and cyanide (connecting Co with W atom) ligands connecting the metal nodes. CoW‐MOF was then converted into W and Co decorated carbon precursor by annealing under inert atmosphere, and subsequently phosphorized into CoWP at 400 °C via a typical phosphorization process, in which NaH_2_PO_2_ decomposed under N_2_ and reacted with the MOF precursor. Presence of sulfur in Co‐MOF, and nitrogen in Co‐MOF and cyanide resulted in dispersion of CoWP in N‐ and S‐doped carbon nanowires (S‐CoWP@S,N‐C) (Figure 7d). Moreover, it was proposed that the MOF synthesis approach offers opportunities for preparation of similar MOF structures by replacing the metals by other transition metals with the same coordination numbers as Co and W (e.g., Ni, Cu replacing Co and Mo replacing W), making it a generic method for designing a number of transition metal incorporated MOF derivatives. On the other hand, it is possible to dope alien transition metal atoms by designing MOF@MOF structures that could integrate dissimilar ligands and metal nodes. Recently, ZIF‐67@CoFe‐PBAs were synthesized by leveraging the Co metal nodes in ZIF‐67 through a partial ligand exchange reaction (Figure 7e).[qv: 68c] When Co‐based ZIF‐67 nanoplates were immersed in K_4_[Fe(CN)_6_] aqueous solution at room temperature, the imidazole ligands of ZIF‐67 were exchanged by the Fe(CN)_6_, giving rise to a steady growth of the CoFe‐PBAs nanoparticles on ZIF‐67 nanoplates. Finally, ZIF‐67@CoFe‐PBAs were chemically transformed into mesoporous Fe incorporated CoP hollow triangular plate arrays (Fe‐CoP HTPAs) by high temperature phosphorization treatment using NaH_2_PO_2_ as the P source. Similarly, M‐doped CoP (M = Ni, Mn, Fe) hollow polyhedron frames (HPFs) were prepared from ZIF‐67 to tailor intrinsic properties of CoP (electronic structure and d‐band center) for electrocatalytic HER.[qv: 68d] Metal precursors (Ni(acac)_2_, Mn(acac)_2,_ and Fe(C_5_H_5_)_2_ with molecular diameter of 5.4, 5.6, and 3.6 Å, respectively) were encapsulated by the ZIF‐67 cages (pore and cage diameter of 3.4 and 11.6 Å, respectively) by a coprecipitation reaction. The encapsulated metal precursors were strongly confined in the framework since the molecular diameters of the metal precursors are smaller than the pore size of ZIF‐67. After posttreatment processes, Ni, Mn, and Fe incorporated CoP HPFs were formed, in which the metal loadings were determined as 0.4, 0.77, and 1.41 wt%, respectively. Although most of the MOF‐assisted transition metal incorporation‐based studies have employed inherent MOF metal as the host and the alien metal as the incorporated metal in the MOF‐derived nanostructures, MOFs can also be tuned to selectively utilize the alien metal as the host in the same MOF derivatives via ion exchange reactions under appropriate conditions.[qv: 68b,69] For example, recently, Su et al. designed Ru‐exchanged Cu‐BTC (HKUST‐1) derivative through an ion‐exchange reaction, where Cu in Cu‐BTC polyhedrons underwent a cation exchange process with Ru when RuCl_3_ solution was added into the parent MOF solution (Figure 7f).[qv: 68b] Taking the advantage of the large pores, open framework structure and weak coordination bonds, high degree of metal replacement was attained with a Cu to Ru elemental ratio of 1:10.65. Subsequently, Ru‐exchanged MOF derivative was decomposed and converted into Cu‐doped with high index faceted RuO_2_ nanoparticles by calcining in air.

### 3.3 Physicochemical Modification of MOF Derivatives

Nanostructures with high surface areas and porosities are promising in both energy and environmental applications. In this sense, physical properties of the MOF derivatives are of particular importance for developing high performance functional materials, which is directly related with the design of parent MOF structures. Chemical coordination affinity of metal nodes to numerous organic ligands in MOFs offers extensive design possibilities, thus allowing exquisite manipulation of both MOFs and MOF‐derivatives properties. Considering the potential effect of metal node and ligand design on the structural and compositional properties of MOF‐derived materials, the relation between the MOF precursors structural properties and MOF‐derivative physicochemical properties has been investigated by judiciously selecting metal clusters and organic linkers at molecular level.^70^ To corroborate this proposition, Zn‐based MOFs with different organic ligands have been particularly designed due to low boiling point of metallic Zn species. Aiyappa et al. studied the effect of ligand characteristics of the parent MOFs on the MOF‐derived carbons by synthesizing Zn‐based MOFs with various organic ligands.[qv: 70c] Carbons transformed from organic ligand containing MOFs exhibited good degree of rigidity and retained the original MOF morphology, while carbons derived from flexible linker containing parent MOFs decomposed in a disordered fashion. Formation of abundant pores due to evaporation of Zn from the carbon phase yielded carbon materials with tunable surface areas depending on the Zn content of parent MOF. Similar phenomenon was reported in Cd‐based MOFs, where carbon materials with tunable surface areas were prepared by evaporating Cd (boiling point = 767 °C) from MOFs, which were synthesized using different ligands.[qv: 70f] Importantly, these studies emphasized the role of tunable carbon materials in energy storage and gas separation processes. Besides the design of MOF‐derived materials via ligand change, it is possible to prepare MOF‐derivatives with controlled particle sizes and surface areas via changing the metal precursor salts. The pioneering work reported by Yamauchi's group demonstrated the effect of metal salt choice on size and surface area of ZIF‐8‐ and ZIF‐8‐derived carbon materials.[qv: 70d] Lower surface area nanosized (≈50 nm) and higher surface area microsized (≈2 µm) ZIF‐8 particles were synthesized by employing Zn(NO_3_)_2_ and Zn(CH_3_COO)_2_ salts, respectively. Formation of ZIF‐8 crystals with different particle sizes was attributed to the nucleation rate difference, which is determined by the solvation rate and interaction of the salt ions.

Apart from the alteration of MOF nodes and linkers to elaborate MOF‐derivatives with superb physical properties, fabrication of robust self‐supported MOF‐derived nanostructures is also an effective way to improve these properties. Integrated self‐supported nanostructures are of particular importance in mass and charge transport related processes since they offer large active areas and facile electrolyte/solution access pathways by eliminating the use of binder and conductive additives that cause undesired nanoparticle aggregation and dead volume problems. Recently, our group employed nickel carbonate hydroxide (NiCH) and cobalt carbonate hydroxide (CoCH) as coordinative templates to initiate nucleation and 3D interpenetrated growth of NiFe–PBAs and CoFe–PBAs, respectively, by a kinetically controlled room temperature crystallization process.[qv: 54a] Specifically, thermal transformation of NiFe–PBA@NiCH with Se powder unveiled tenaciously bridged and robust selenide phase elaborated with rich unsaturated sites due to dehydroxylation/carboxylation of underlying carbonate hydroxide substrate during the thermal decomposition, indicating the key roles of substrate as nucleation, growth and defect generation medium. The as‐designed interpenetrating structure featured abundant unsaturated atomic sites, porous morphology, high conductivity, and high surface area, and thus served as an efficient bifunctional electrocatalyst for overall water splitting.

## 4 Energy‐Related Applications

### 4.1 Electrochemical Energy Conversion

Advance in electrochemical technologies, including water‐splitting, metal–air batteries and fuel cells, is an indispensable requirement to meet the ever‐growing future energy demand since they offer sustainable and clean energy that could diminish the fossil‐fuel dependency and global warming.^71^ Fundamentally, advancement in these technologies is directly related to the development of electrocatalysts that play decisive role on the performance of core electrochemical reactions, such as the HER, oxygen evolution reaction (OER), ORR, etc., taking place in the electrochemical energy conversion devices. However, the electrochemical reactions (specifically, OER and ORR) generally exhibit sluggish reaction kinetics, necessitating the use of electrocatalysts with high activity yet long‐term stability to overcome the high energy barriers at low overpotentials. Until now, MOFs have gained revived interest in the field of electrocatalysis. Morphological engineering of MOFs toward synthesis of complex nanostructured electrocatalysts (e.g., single‐shelled hollow structures, multishelled hollow structures, yolk–shell structures) have been widely reported so far.^72^ Abundant accessible active sites and facile mass/charge transport pathways have endowed MOF‐based electrocatalytic materials with exceptional electrochemical properties.^72^ Similarly, intrinsic atomic and compositional engineering of MOFs have also been claimed to boost electrocatalytic performance. At this point, recent progress should be provided about the essential insights on the atomic‐ and molecular‐level structural engineering strategies to improve electrocatalytic activity of MOFs and MOF derivatives, which will be discussed in this section.

*HER*: H_2_ is regarded as an ideal future energy carrier since it exhibits a high gravimetric energy density with zero carbon emission.^73^ Hence, the design of electrocatalytic materials for hydrogen evolution and understanding of the underlying mechanisms play pivotal roles on advancing the future energy systems. Theoretically, each HER mechanism is initiated by the Volmer reaction for electrochemical adsorption of hydrogen atom (H^+^), which is then followed by H_2_ desorption step via either Heyrovsky reaction or Tafel reaction. In acidic conditions, the HER activity is mainly determined by the interaction strength between the catalytic surface and H^+^, which is quantified by the adsorption free energy of hydrogen (Δ*G*_H_). Δ*G*_H_ should be sufficiently strong for facile surface adsorption and desorption processes, corresponding to a value close to 0 on the volcano plots as suggested by the Sabatier principle.^74^ Pt is located close to the apex of the volcano plot with an almost zero Δ*G*_H_, thus it is considered to be the benchmark HER electrocatalyst.[qv: 74b,d] Although electrocatalysts are commonly evaluated in acidic electrolytes for HER, alkaline conditions are also very desirable considering the milder electrolyte environment and high activity of OER electrocatalysts in basic electrolytes in case of overall water splitting. However, in alkaline conditions, the origin of electrocatalytic activity is correlated not only with Δ*G*_H_ but also with dissociation kinetics of water, which does not exist in acidic conditions.^75^ Thus, highly active electrocatalysts in acidic electrolytes, such as benchmark Pt, may not achieve similar activity in alkaline conditions. In this regard, structural and electronic properties (crystallinity, d‐band center position, work function, Fermi level, etc.) of electrocatalysts can be rationally tuned manipulate the adsorption energetic of surface and activation energies.

**Figure 8:**
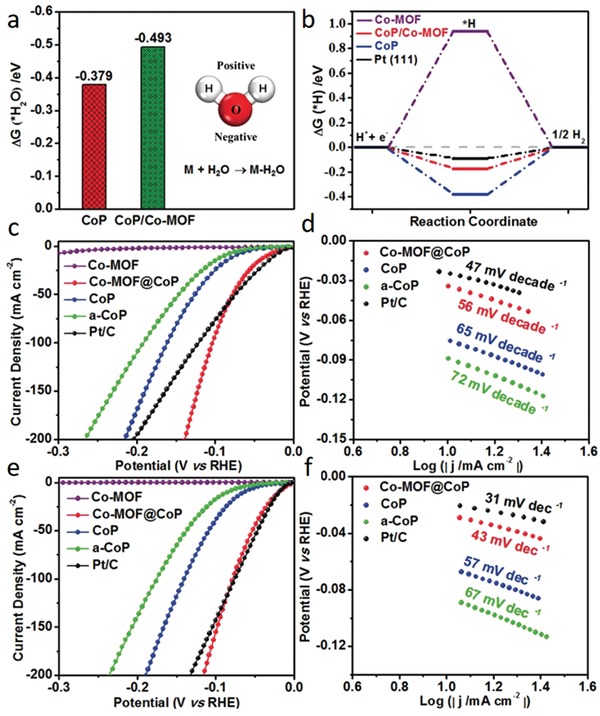
The calculated free‐energy diagram for a) water and b) hydrogen adsorption on different catalysts. c) HER polarization curves and d) corresponding Tafel plots of the catalysts in 1 m KOH. e) HER polarization curves and f) corresponding Tafel plots of catalysts in 0.5 m H_2_SO_4_. a–f) Reproduced with permission.[qv: 76g] Copyright 2019, Wiley‐VCH.

Both pristine MOFs and their derivatives have been engineered to optimize the adsorption free energy of reaction intermediates.[qv: 48f,50,54a,63e,66a,76] Liu and co‐workers reported that the optimized H_2_O adsorption energy (∆*G*_H2O_) and ∆*G*_H_ could be obtained by modulating charge distribution via partial transformation of MOF into phosphide, which boosts the activity of the phosphide phase and makes it a highly efficient pH‐universal electrocatalytic material.[qv: 76g] Partial transformation of N‐containing Co‐MOF into CoP with intact interface over N atoms stimulates interfacial electron transfer to the Co atoms in CoP due to stronger electronegativity of N atoms in Co‐MOF. The resulting hybrid hosts Co atoms with altered electronic states of d‐orbital, entailing more optimal ∆*G*_H2O_ and ∆*G*_H_ (**Figure** **8**a,b). As a result, Co‐MOF@CoP exhibited excellent HER performance over the full pH range by requiring overpotentials of only 49, 34 and 27 mV in 1 m PBS, 1 m KOH, and 0.5 m H_2_SO_4_ to reach −10 mA cm^−2^, respectively (Figure 8c,e). Moreover, favorable kinetic process of the Co‐MOF/CoP at different pH values was further confirmed by low Tafel slopes of 63, 56, and 43 mV dec^−1^ in 1 m PBS, 1 m KOH, and 0.5 m H_2_SO_4_, respectively, which are comparable to Pt/C (Figure 8d,f). Similarly, taking the advantage of inherent Co atoms, low‐boiling point Zn atoms and high N content, ZIF‐67@ZIF‐8@rGO was rationally designed to obtain 0D Co nanoparticles tightly encapsulated by in situ formed 1D‐N‐doped CNTs, forming Co@N‐CNTs.^50^ DFT calculations unveiled that Co@N‐CNTs@rGO assures optimal hydrogen atom adsorption with a ∆*G*_H_ value near to zero (0.16 eV). Further theoretical calculations revealed that the Co/C contact and nitrogen dopant transforms the carbon layer into n‐type‐doped to modify the π‐conjugated system of C and redistribute the charge density of the Co@C‐H system. The optimized electrocatalyst afforded a low overpotential (108 mV at −10 mA cm^−2^) and a high stability with a low Tafel slope (55 mV dec^−1^) in 1 m KOH solution.

Recently, MOF‐derived single atom catalysts have been regarded as a new frontier in the electrocatalyst platform since they offer advantages of high activity and durability because of the maximum atom utilization, strong electronic, and covalent interaction between the monodispersed metal atoms and inherent carbon/nitrogen atoms, and high electrical conductivity.[qv: 63e,77] Taking UiO‐66‐NH_2_ as an example, Chen and co‐workers designed a stable and highly active HER electrocatalyst by transforming W impregnated MOF into N‐doped carbon decorated with single tungsten atoms (W‐SAC). W‐SAC required a low overpotential (85 mV) to reach a current density of −10 mA cm^−2^, and exhibited small Tafel slope (53 mV dec^−1^), high turnover frequency (6.35 s^−1^ at the overpotential of 120 mV) and robust operation (no appreciable activity degradation after 10 000 CV cycles), which are close to that of the commercial Pt/C in 0.1 m KOH. DFT calculations indicated that C atom close to N atom ensures the most favorable hydrogen atom adsorption site with the lowest ∆*G*_H_ value of 0.033 eV. Differential charge density (Δρ) and DOS calculations further clarified that single W atoms induce charge transfer through the W d‐orbital and lead to an increase in the electron density on the C atoms, thus promoting stronger proton adsorption on the catalytic surface that eventually lowers the ∆*G*_H_.

In the aforementioned literature, MOFs are treated at high temperatures to be partially or entirely transformed into MOF‐derivatives or MOF/MOF‐derivative hybrids. Indeed, pristine MOFs can be well utilized as electrocatalytic materials by leveraging the abundant inherent molecular metal centers, high porosities, and high surface to volume ratios.^78^ However, MOFs generally exhibit poor electrical conductivities (≈10^−10^ S m^−1^), which hinder their efficiencies in essential charge transfer kinetics during the electrocatalytic processes, thereby lowering their overall catalytic activity.[qv: 78c,79] In this regard, Duan et al. rationally designed an ultrathin (thickness ≈ 3.5 nm) yet large (lateral size > 100 nm) and conductive (1 ± 0.2 × 10^−3^ S m^−1^) 2D NiFe‐MOF nanosheet structure to manifest greater fraction of catalytic active sites that is hardly accomplishable in 3D counterparts, as supported by electrochemically active surface measurements.[qv: 78c] High conductivity of the 2D NiFe‐MOF was ascribed to the defective 2D nanostructuring process since it inherently entails topologically defects in form of vacancies due to terminated coordination in ultrathin 2D structure, which modifies the carrier concentration of metal octahedral units in the MOF and allows a superior electron transfer process, thus improving the electrical conductivity. 2D NiFe‐MOF nanosheets achieved a good HER activity with a low overpotential of 134 mV at the current density of 10 mA cm^−2^ and a high turnover frequency (2.8 s^−1^ at the overpotential of 400 mV) in 0.1 m KOH.

*OER*: OER plays a dominant role in determining the effectiveness of key electrochemical energy storage and conversion technologies, including fuel cells, metal–air batteries, and water electrolyzers. However, water oxidation to molecular oxygen possesses a four‐step complex reaction pathway with sluggish reaction kinetics on most of the electrocatalysts, significantly hampering further development of these technologies.^80^ Theoretically, from thermodynamic point of view, an ideal catalyst should possess a ΔG of 1.23 V at standard conditions (pH 0, T = 298.15 K), while real catalysts exhibit larger ΔG values since the OER process is basically hindered by the nonoptimal OH*→O*→ OOH* intermediates adsorption/desorption energies, resulting in large overpotentials and unfavorable reaction kinetics.[qv: 80a] In this regard, MOF structures have been ideal platforms to investigate the electronic properties of various adsorption sites through theory and experiments due to their highly tunable bonding arrangements and diverse coordination network constituents, which could unveil structure–OER performance relationships at the atomic level.^81^

**Figure 9:**
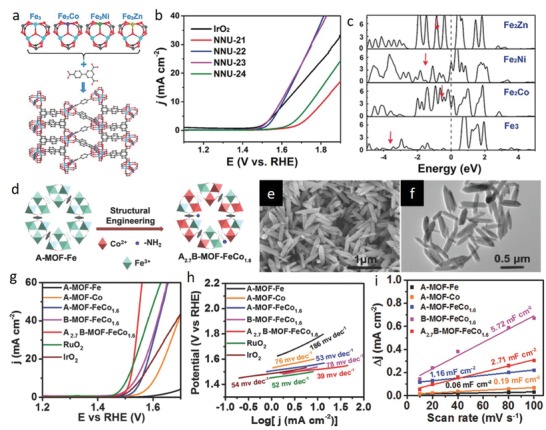
a) Framework of NNU‐21‐24 connected by trinuclear metal clusters and tridentate carboxylate ligand. b) OER polarization curves recorded in 0.1 m KOH. c) Projected density of states for NNU‐21‐24. a–c) Reproduced with permission.[qv: 81g] Copyright 2018, Wiley‐VCH. d) Illustration of the design strategy for heterogeneity A_2.7_B–MOF–FeCo_1.6_ catalyst preparation. e) SEM and f) TEM images of A_2.7_B–MOF–FeCo_1.6_. g) OER polarization curves obtained at a scan rate of 5 mV s^−1^. Corresponding h) Tafel plots and i) capacitive currents as a function of the scan rate for various catalysts measured in 1 m KOH. d–i) Reproduced with permission.[qv: 81a] Copyright 2018, Wiley‐VCH.

Particularly, multitopic MOFs, MOFs with different type of metal centers, have been designed to explore the bimetallic/trimetallic coupling effect on the electronic structure of the adsorption sites for OER.[qv: 81b,c,g] Wang et al. designed a series of Fe_3_(µ3‐O)(CH_3_COO)_6_(H_2_O)_3_‐based metallic clusters by substituting an iron atom in Fe_3_ by a secondary metal atom (M: Fe, Co, Ni, and Zn) with a similar radius.[qv: 81g] The bimetallic clusters were then bridged by biphenyl‐3,4'5‐tricarboxylic acid (BPTC) ligands to obtain isostructural Fe_3_‐BPTC (NNU‐21), Fe_2_Co‐BPTC (NNU‐22), Fe_2_Ni‐BPTC (NNU‐23), and Fe_2_Zn‐BPTC (NNU‐24) MOFs (**Figure** **9**a). All the pristine heterometallic Fe‐based MOFs realized better OER activity than the corresponding monometallic counterparts (Figure 9b). Among them, Fe_2_Ni‐BPTC (NNU‐23) exhibited the best OER performance with the lowest overpotential (365 mV at 10 mA cm^−2^), smallest Tafel slope (72.2 mV dec^−1^), and highest ECSA (5.10 mF cm^−2^) and TOF (0.03 s^−1^ at 400 mV) values in 0.1 m KOH. It was further corroborated by the spin‐polarized DFT calculations that compared with Fe_3_ cluster, Fe_2_M clusters strengthen the weak adsorption of oxygen intermediate (O*) on the active Fe site and lower the Δ*G*_O*_. Moreover, DOS calculations further provided a deeper insight into the effect of secondary metal atom substitution on catalytic performance by revealing that d‐band center of the Fe_2_Co, Fe_2_Ni, and Fe_2_Zn clusters are closer to the Fermi level than that of Fe_3_ cluster, which enhances the binding interaction between the adsorbate and catalyst (Figure 9c). Besides the type of secondary metal atom, the effect of loading content on the synergistic interactions of the heteroatoms and the electronic structure of adsorption surfaces was also investigated for OER using pristine MOFs and its derivatives.[qv: 81b,c] For example, Zhou et al. designed isostructural MOFs, [NH_2_(CH_3_)_2_][M_3_(µ_3_‐OH)(H_2_O)_3_(BHB)], by altering the composition of trinuclear heterometallic carboxylate clusters (M_3_: Co_3_, Co_2_Ni, CoNi_2_, Ni_3_).[qv: 81c] Compared with Co_2_Ni‐MOF and monometallic MOFs (Ni_3_‐MOF and Co_3_‐MOF), CoNi_2_‐MOF displayed a superior OER performance with an ultralow overpotential requirement of 240 mV to afford a current density of 10 mA cm^−2^ in 0.1 m KOH, being even lower than that of the benchmark RuO_2_. Moreover, the Tafel slope of the CoNi_2_‐MOF (58 mV dec^−1^) is smaller than that of the Co_2_Ni‐MOF (81 mV dec^−1^), Ni_3_‐MOF (127 mV dec^−1^), Co_3_‐MOF (92 mV dec^−1^), and RuO_2_ (62 mV dec^−1^). On the basis of electrochemical tests, substituting one Ni atom by Co in trinuclear monometallic Ni carboxylate clusters (Ni_3_‐MOF) improves ECSA and electrical conductivity. From the theoretical point of view, the excellent activity of CoNi_2_‐MOF was ascribed to the shift of the d‐band center to a higher energy level and optimized intermediate adsorption with the incorporation of Ni metal atoms. These examples clearly highlight the prominent function of atomically designed MOFs on the fundamental understanding of the energetics of the OER.

Apart from the metal nodes, the versatility of the organic linkers also facilitated the research on the synergistic interaction of mixed linkers. In this respect, a heterogeneity MOF A_2.7_B‐MOF‐FeCo_1.6_ was designed at the atomic and molecular level by introducing mixed metal (Fe and Co) and organic linkers (A: terephthalic acid and B: 2‐aminoterephthalic acid) into the original MIL‐88B (Figure 9d–f).[qv: 81a] The A_2.7_B‐MOF‐FeCo_1.6_ electrode displayed a very low overpotential (288 mV dec^−1^ to drive 10 mA cm^−2^), a small Tafel slope (39 mV dec^−1^), a high double‐layer capacitance (*C*_dl_) (2.71 mF cm^−2^) and a high TOF (0.46 s^−1^ at an overpotential of 300 mV) in 1 m KOH, exceeding the OER activity of A‐MOF‐FeCo_1.6_ and B‐MOF‐FeCo_1.6_ (Figure 9g–i). Spectroscopic and first‐principle calculations further unveiled that the electron density in the 3d orbital of Co atoms in A_2.7_B‐MOF‐FeCo1.6 increases with linker substitution and becomes more delocalized, which can efficiently trigger the rate determining step for the formation of OOH* intermediates.

Defect engineering of MOFs is another powerful technique to manipulate the OER catalytic activity.[qv: 54a,81d,e] For example, in the pioneering work, Zhao et al. demonstrated that ultrathinning of NiCo‐MOF endows the 2D structure with abundant coordinatively unsaturated sites, eventually leading to a substantial improvement in OER activity.[qv: 81d] They reported the synthesis of ultrathin NiCo bimetal–organic framework nanosheets (NiCo‐UMOFNs) from benzenedicarboxylic acid (BDC) type linkers. The electrode displayed an excellent electrocatalytic OER performance with an overpotential of 189 mV at a current density of 10 mA cm^−2^, a Tafel slope of 42 mV dec^−1^ and TOF of 0.86 s^−1^ (at 300 mV overpotential) in 1 m KOH solution, exceeding the performance of its isostructural Co‐UMOFNs and Ni‐UMOFNs counterparts and bulk NiCo‐MOFs. EXAFS analysis, XANES simulation, and DFT calculation further suggested that the high electrocatalytic activity originates from the coordinatively unsaturated nature of surface metal sites on NiCo‐UMOFNs surfaces due to terminated BDC ligands.

*ORR*: Proton‐exchange‐membrane fuel cell (PEMFC), which directly converts the chemical energy of hydrogen and oxygen into electricity, holds great promise as an energy conversion device due to its high conversion‐efficiency, zero carbon footprint, high reliability, simple design, and portable applications.^82^ While the hydrogen oxidation reaction (HOR) taking place at the anode side is rather fast and efficient, ORR at the cathode generally suffers from sluggish reaction kinetics, high overpotential, and low electrocatalyst stability, resulting in considerable loss in overall harvestable energy.^82^, ^83^ Platinum group metal (PGM) catalysts are regarded as the benchmark ORR electrocatalysts, yet they are the costliest component of the cell accounting for as much as 34% of the total cost of the PEMFC according to a U.S DOE estimation.^82^, ^83^ Recent studies suggested that low‐cost carbonaceous electrocatalysts co‐doped with transition metals and nitrogen (M–N–C electrocatalysts, where M: Fe, Co, Mn, Cu, or Ni) can be as active as PGM electrocatalysts due to favorable oxygen adsorption and O—O bond breakage characteristics, and they exhibit higher stability.[qv: 54d,84] Traditional strategy for preparation of M–N–C electrocatalysts includes pyrolysis of physically mixed carbon, nitrogen, and transition metal precursors. However, this process is usually uncontrollable over molecular structure and morphology, leading to formation of metallic clusters/NPs, uneven distribution of elements and aggregated morphologies, which greatly reduces not only the accessible surface area but also the number of active catalytic sites.[qv: 54d,85] In this respect, MOFs are exceptional precursors for controllable synthesis of M–N–C electrocatalysts with uniform elemental distribution and abundant active catalytic sites.

**Figure 10:**
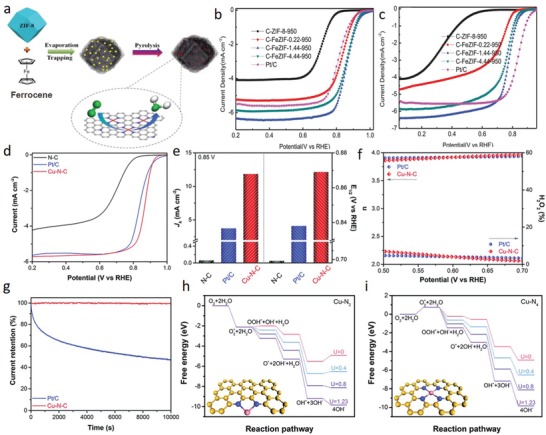
a) Synthesis strategy for preparation of Fe–N–C catalysts. b,c) ORR polarization curves of various catalysts in O_2_‐saturated b) 0.1 m KOH and c) 0.1 m HClO_4_. a–c) Reproduced with permission.^86^ Copyright 2019, Wiley‐VCH. d) ORR polarization curves and e) kinetic currents (*J*_K_) (at 0.85 V) and half‐wave potentials for Cu–N–C, N–C and Pt/C catalysts in O_2_‐saturated 0.1 m KOH solution. f) Electron transfer number (*n*) and H_2_O_2_ yield for Cu–N–C and Pt/C. g) *i*–*t* curves of Cu–N–C and Pt/C for 10 000 s at a constant potential. Calculated free‐energy diagram for ORR on h) Cu–N_2_ and i) Cu–N_4_ active sites. The inset structures represent the optimized structures of Cu–N_2_ and Cu–N_4_. The yellow, blue, and light pink balls represent carbon, nitrogen, and copper atoms, respectively. d–i) Reproduced with permission.^65^ Copyright 2018, The Royal Society of Chemistry.

Among the M–N–C electrocatalysts, Fe–N–C electrocatalysts elaborated with Fe–N_4_ site are the best performing ones, exhibiting ORR activities comparable to Pt/C in both alkaline and acidic electrolyte.^84^ Chen et al. investigated the role of FeN_4_ on ORR activity by performing complimentary control experiments using MOF precursor and first‐principle calculations.[qv: 64a] It was demonstrated that high activity originates from the facile electron transfer from single Fe atoms in the specific FeN_4_ configuration, accelerating the rate determining *OH to OH^−^ formation step on Fe–N–C catalysts. To synthesize Fe–N–C electrocatalysts with uniform distribution and high Fe content, ZIFs and MOFs have been rationally engineered by incorporating Fe atoms through intrinsic ionic coordination and pore confinement strategies, or postprocessed to trap Fe atoms with the help of intrinsic N atoms.[qv: 61c,62,64a,86] For instance, Fe–N–C catalysts were synthesized via coordinating Fe atoms with ligands in Zn‐based imidazolate framework and subsequently converting them into porous Fe‐ and N‐doped carbon materials through thermal activation.[qv: 61c] The optimized catalyst exhibited a half‐wave potential (*E*_1/2_) of 0.85 V in 0.5 m H_2_SO_4_, only 30 mV lower than Pt/C catalysts (0.88 V in 0.1 m HClO_4_) with a negligible H_2_O_2_ yield (less than 1%). Deng and co‐workers demonstrated a gaseous doping strategy to prepare N‐doped carbon polyhedron catalysts embedded with single Fe atoms for Fe–N–C catalysts (**Figure** **10**a).^86^ It was reported that single Fe atoms existing in the form of FeN_4_ configuration (C‐FeZIF‐1.44‐950) trigger the ORR activity of N–C in both alkaline and acidic electrolyte. *E*_1/2_ of 0.864 V was achieved in 0.1 m KOH that is 50 mV higher than that of commercial Pt/C, and *E*_1/2_ of 0.78 V was recorded in 0.1 m HClO_4_, 60 mV less than that of Pt/C catalyst (Figure 10b,c).

ORR activity of M–N–C electrocatalysts generally follows the trend of M: Fe > Co > Cu > Mn in both alkaline and acidic electrolytes. Although Fe–N–C has been the most promising, M–N–C catalysts with Co, Mn and Cu have recently gained interest due to high catalytic activity of Fe toward Fenton reactions, where H_2_O_2_ is decomposed and side products such as hydroxyl and hydroperoxyl radicals are generated causing to a decrease in ORR activity of the Fe–N–C catalyst and degradation in PEMFC polymer.^87^ For example, a series of Co–N–C catalysts, containing Co species in form of NPs (Co‐NPs@NC), atomic clusters (Co‐ACs@NC) and single atoms (Co‐SAs@NC) in N‐doped carbon, was proposed to investigate the size‐dependent catalytic properties via rationally adjusting the spatial concentration of Co and Zn atoms in BMOF.[qv: 61e] Electrochemical tests revealed that Co‐SAs@NC exhibits superior catalytic activity (*E*_1/2_: 0.82 V in 0.1 m KOH), faster reaction kinetics (kinetic current density: 15.2 mA cm^−2^) and lower peroxide generation (below 3%) than Co‐NPs@NC and Co‐ACs@NC in alkaline media. Li and co‐workers studied transformation of CuBTC MOF into Cu–N–C via external nitrogen stabilization, and investigated the origin of ORR activity by analyzing the molecular structure of Cu–N–C via spectroscopic characterizations and DFT calculations.^65^ Cu–N–C exhibited better stability and a more positive *E*_1/2_ (0.869 V) than Pt/C (0.838 V) with a similar H_2_O_2_ yield and electron transfer number (n: 3.97), indicating excellent performance of the Cu–N–C toward ORR in 0.1 m KOH (Figure 10d–g). DFT calculations further revealed that Cu–N_2_ offers favorable sites for optimum O_2_ and OOH adsorption; while Cu–N_4_ and Cu‐free N–C possesses relatively weak O_2_ and OOH adsorption sites, which is dissimilar to the Fe–N–C and Co–N–C catalysts, in which M‐N_4_ has been mostly reported to be the most optimal ORR‐active site (Figure 10h,i). Recently, a MOF‐based two‐step Mn doping and adsorption approach was proposed to prepare atomically dispersed Mn sites in N–C electrocatalysts with high density as a promising alternative to traditional Mn–N–C synthesis routes that lead to Mn aggregation in the carbon framework.^87^ Mn–N–C exhibited an *E*_1/2_ (≈0.80 V), which is higher than Co–N–C, comparable to Fe–N–C and 60 mV less than that Pt/C in 0.5 m H_2_SO_4_. DFT calculations indicated that MnN_4_C_12_ is the most optimal site that thermodynamically and kinetically promotes the four‐electron transfer pathway with optimum activation energies.

**Figure 11:**
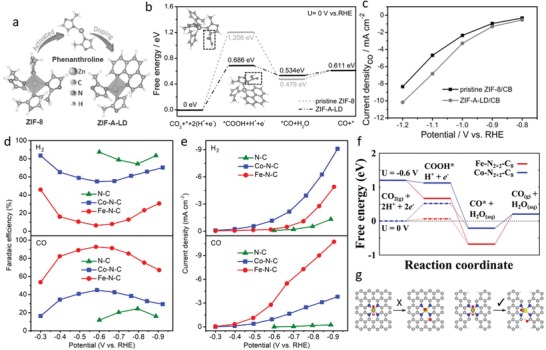
a) ZIF‐A‐LD synthesis strategy. b) Calculated Gibbs free energy diagrams for CO_2_ electroreduction to CO on the sp^2^ C atom sites in imidazole ligand of pristine ZIF‐8 and ZIF‐A‐LD. c) CO partial current densities measured in CO_2_‐saturated 0.1 m KHCO_3_ solution for pristine ZIF‐8/CB and ZIF‐A‐LD/CB. a–c) Reproduced with permission.[qv: 89c] Copyright 2019, Wiley‐VCH. d) FEs and e) partial current densities for N–C, Co–N–C and Fe–N–C catalysts measured in CO_2_‐saturated 0.1 m KHCO_3_. f) Calculated Gibbs free energy diagrams for CO_2_ electroreduction to CO on Fe–N–C and Co–N–C under an applied electrode potential (*U*) of 0 V and –0.6 V. g) Initial and final states of CO_2_RR steps on M–N–C active sites. d–g) Reproduced with permission.^90^ Copyright 2018, American Chemical Society.

*CO_2_ Reduction Reaction*: Electrochemical CO_2_ reduction reaction (CO_2_RR) offers a promising opportunity to convert the most detrimental greenhouse gas into useful renewable fuels and value‐added chemicals, greatly contributing to the environmental remediation and energy generation. It follows energetically and kinetically unfavorable multistep processes, including two‐, four‐, six‐, eight‐, or twelve‐electron pathways for production of a variety of carbon‐based compounds such as CO, HCOOH, HCHO, HCOOH, CH_3_OH, CH_4_, C_2_H_4,_ and C_2_H_5_OH. To date, active sites of pristine MOFs and MOF‐derivatives for CO_2_RR have been investigated and modulated to synthesize electrocatalysts with high Faradaic efficiency (FE), selectivity, and long‐term stability. Among pristine MOFs, metalated porphyrinic MOFs have been demonstrated to possess intrinsic active sites toward CO_2_RR.^88^ In the pioneering study, Kornienko et al. synthesized a series of MOFs films by designing the porphyrin units with different metal centers [Al_2_(OH)_2_TCPP‐M', where M': Zn, Cu, and Co].[qv: 88a] Among them, MOF with cobalt‐metalated porphyrin units exhibited the highest activity for formation of CO with a FE of 76%, Tafel slope of 165 mV per decade, a per‐site turnover number (TON) of 1400 and remarkable stability over 7 h. The same units with modified the inorganic backbone [M_2_(OH)_2_TCPP‐Co, where M: Al and In] still showed a remarkable CO_2_ conversion activity, signifying that porphyrin units are the active sites. In situ spectroelectrochemical studies further suggested that during the electrochemical process, Co(II) centers are reduced to Co(I) centers, which subsequently react with CO_2_ to form CO, indicating that major CO_2_RR active sites in porphyrin units are the Co(I) metal centers. While redox‐accessible metal centers have been reported to be the catalytic active sites for CO_2_RR in most of the MOFs, ligands have been reported to be optimal active sites in Zn‐based MOFs due to fully occupied 3d orbital of Zn (II).^89^ Jiang and co‐workers have recently investigated the active sites for CO_2_RR by designing Zn‐based MOFs (ZIF‐8, ZIF‐108, ZIF‐7, and SIM‐1) with the same SOD (sodalite) topology and different organic ligands.[qv: 89a] In situ X‐ray absorption spectroscopy measurements and DFT calculations revealed that imidazolate ligands coordinated with Zn (II) centers are the active sites responsible for CO_2_ conversion. Considering this, a molecular design strategy was proposed to boost the activity of catalytic sites of ZIF‐8 via electron‐donating ligand doping.[qv: 89c] Taking the advantage of labile bonds, a strong electron‐donating molecule of 1,10‐phenanthroline was introduced to the vacant Zn sites generated by thermal activation process of ZIF‐8 (ZIF‐A), the ligand doped product is denoted as ZIF‐A‐LD (**Figure** **11**a). Spectroscopic studies and DFT calculations further elucidated that charge transfer is realized from the ligand to the imidazole linker, entailing a charge density increase on the sp^2^ C atom from 3.702 to 4.884e^−^, and Gibbs free energy decrease from 1.2016 to 0.686 eV for formation of *COOH on the same carbon atom sites in imidazole ligand of ZIF‐A‐LD compared with that in pristine ZIF‐8 (Figure 11b). As a result, partial current density for CO catalyzed by ZIF‐A‐LD/CB (CB: carbon black, to improve electrical conductivity) reached a higher value than that by pristine ZIF‐8/CB (Figure 11c). Moreover, ZIF‐A‐LD‐CB generated CO with a high FE of 90.57% at −1.1 V and exhibited a Tafel slope of 198 mV dec^−1^, which is superior to the CO_2_RR activity of pristine ZIF‐8/CB.

Apart from pristine MOFs, MOF‐derived atomically dispersed M‐N‐C materials have been recently regarded as a promising class of CO_2_RR electrocatalysts.[qv: 64b,90] For instance, Fe–N_4_ and Co–N_4_ sites were generated by rationally designing the metallic nodes of pristine ZIF before carbonization process. Fe–N_4_ sites were reported to possess a high current density and FE (93%) for the production of CO, which is intrinsically more active than Co‐N_4_ (FE: 45%) and N–C (FE: 20%) sites (Figure 11d,e). DFT calculations suggested that OH^−^ is strongly adsorbed on C atoms with dangling bonds and *CO is adsorbed on M centers during C=O bond cleavage, making CO formation more favorable then H_2_ formation (Figure 11f,g).^90^ In a similar study investigating the effect of Co centers coordination number on CO_2_RR activity, Co–N–C catalysts with Co–N_2_, Co–N_3,_ and Co–N_4_ were prepared by engineering of MOF‐derivatives during pyrolysis.[qv: 61b] Co–N_2_ attained the best catalytic activity with the FE for CO formation as high as 94%, a current density of 18.1 mA cm^−2^ at an overpotential of 520 mV and a TOF value of 18200 h^−1^ at ‐0.63 V, exceeding the activity of Co‐N_3_ (FE: 63%) and Co‐N_4_ (FE: <5%). The insight into the electrocatalytic CO_2_ conversion process gained by DFT studies revealed that Co‐N_2_ sites ensures strong interaction with the CO_2_^.−^ intermediate and inhibits H_2_ formation.

Besides the electrocatalytic CO_2_ reduction, photocatalytic CO_2_ reduction, CO_2_ organic transformation (e.g., cycloaddition with epoxides, oxidative carboxylation of olefins, and carboxylation of terminal alkynes) and CO_2_ hydrogenation are alternative strategies for conversion of CO_2_ into value‐added products and fuels over pristine MOFs and MOF‐derived catalysts. Extensive efforts have been devoted to exploring various atomic and molecular design strategies, including introduction of defects and incorporation of coordinatively unsaturated metal nodes and functional linkers, to improve frameworks' CO_2_ affinity and CO_2_ conversion capability.^91^ MOF‐based catalysts possessing abundant CO_2_ accessible pores that are elaborated with Lewis/Brønsted acid sites as well as basic sites have exhibited promising CO_2_ conversion performances.^92^ However, comprehensive discussion of these alternative strategies is beyond the scope of this review and readers can refer to other review papers on CO_2_ capture and conversion.^91^, ^93^

### 4.2 Electrochemical Energy Storage

**Figure 12:**
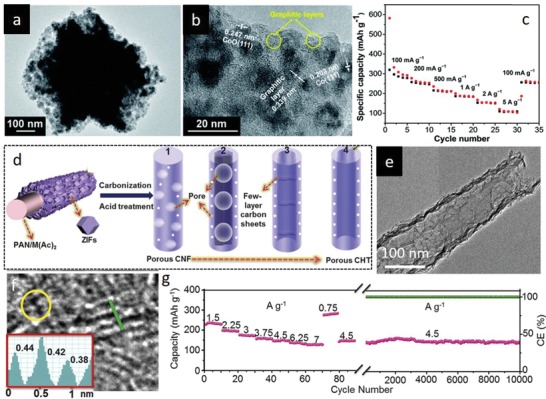
a) TEM and b) HRTEM images of an individual Ni‐doped Co/CoO/NC dodecahedron hybrid. c) Rate capability at various current densities for Ni‐doped Co/CoO/NC. a–c) Reproduced with permission.[qv: 44f] Copyright 2017, The Royal Society of Chemistry. d) Schematic illustration showing the formation of N‐doped porous carbon hollow tubule (CHT). e) TEM and f) HRTEM images of N‐doped porous CHT, inset shows the d‐spacing line‐profile and yellow circle encloses the existence of curvature and defects. g) Rate capability at various current densities and cycling performance at 4.5 A g^−1^ over 10 000 cycles of N‐doped porous CHT. d–g) Reproduced with permission.[qv: 94b] Copyright 2017, Elsevier.

Rechargeable batteries, including lithium‐ion batteries (LIBs), sodium‐ion batteries (SIBs), and lithium–sulfur (Li–S) batteries, have been emerging energy storage options due to revival in portable electronic devices and smart grid technology. The specific capacities and redox potentials of the anode and cathode materials have been the key features governing the performance of batteries. Particularly, development of anode materials for LIBs and SIBs has been an ongoing effort to meet the market energy and power density requirements as the conventional graphite anode possesses a low theoretical capacity (372 mAh g^−1^). Besides, small interlayer spacing of graphite anodes has been a predominant bottleneck in SIBs limiting the insertion of larger‐ionic‐radii Na^+^ ion compared with Li^+^ ion in LIBs.^94^ To this end, pristine MOFs have been studied as potential anode candidates due to their high surface areas, intrinsic porous structure with large size cages/channels and versatility for electrochemically active functional group incorporation and defect introduction.^95^ Yet, insulating nature and poor stability of pristine MOFs have emerged as the main deterrence factors. Nevertheless, transformation of rationally engineered pristine MOFs into transition metal oxides, transition metal chalcogenides and carbon‐based materials with unique porous structures, complex hollow morphologies and defective lattice has offered considerably high specific capacities and stabilities.[qv: 44f,54e,94–96] In particular, bimetallic cobalt‐ and iron‐based oxides have been developed from pristine MOFs modulated via heteroatom doping strategy (e.g., ZIF‐67 (secondary Ni and Zn), Co‐BTC (secondary Ni), PBA, MOF‐5 (secondary Fe), Fe_2_M–MOFs (secondary Zn, Co, Ni, and Mn), etc.) to leverage the synergistic interactions.[qv: 44f,96] For example, Yamauchi's group demonstrated that microporous Ni‐doped Co/CoO/N‐dopedcarbon (NC) hybrid derived from Ni‐Co‐ZIF (**Figure** **12**a,b) shows an excellent electrochemical performance when used as an anode material for SIBs benefitting from the synergistic effects of Ni and N heteroatoms and carbon matrix.[qv: 44f] It was reported that: i) defect sites introduced by Ni and N heteroatom dopants provide higher electrical conductivity and additional electrochemical active sites for reversible Na^+^ ion insertion/removal and ii) microporous carbon network emerged during decomposition of MOF organic linker stabilizes the Co/CoO species, facilitates Na^+^ ion transportation and enhances the structural integrity. Stemming from this unique structure and chemical composition, Ni‐doped Co/CoO/N‐doped carbon (NC) hybrid manifested a good rate performance with a high discharge capacity of 307 mAh g^−1^ at a current density of 100 mA g^−1^ (Figure 12c). Moreover, a remarkable capacity retention as high as 87.5% was achieved after 100 cycles at a high current density of 500 mA g^−1^. Similarly, Chen et al. proposed a BMOF (Zn/Co)‐based calcination and self‐etching strategy to prepare defective N‐doped carbon hollow tubules (CHTs) (Figure 12d) with large interlaying spacing to ensure facile Na^+^ ion transport.[qv: 94b] Decomposition of the atomically designed BMOF and self‐etching of the inner carbon core accompanied by the reduction of Zn and Co at elevated temperature revealed a graphitic carbon structure elaborated with micro/mesopores, extended interlayer spacing, extended lattice defects and N‐doping (Figure 12e,f). By a virtue of these merits, the CHTs exhibited a remarkable rate capability with specific capacities of around 238, 200, 175, 157, 147, 138, and 128 mAh g^−1^ at current densities of 1.5, 2.25, 3, 3.75, 4.5, 6.25, and 7 A g^−1^, respectively, and excellent cycling stability without any capacity fading as presented in Figure 12g.

## 5 Environmental Applications

### 5.1 Gas Storage and Separation

With growing concerns over global warming, gas storage, and separation have become a pressing issue for environmental protection. Considering the porosity and compositional tunability of MOFs, their application as sorbents has attracted substantial research interest. Key separation targets include carbon dioxide (a significant contributor to the greenhouse effect), whereas the storage of hydrogen and methane is considered cornerstone technologies to promote more widespread adoption of clean energy.^97^ These represent complementary efforts toward a global transition to a low‐carbon economy for more sustainable economic development. In addition to this, the replacement of cryogenic distillation for the separation of valuable light hydrocarbons (e.g., ethylene/ethane, propylene/propane) is significant for improving the energy efficiency of petroleum‐based industries.^98^

**Figure 13:**
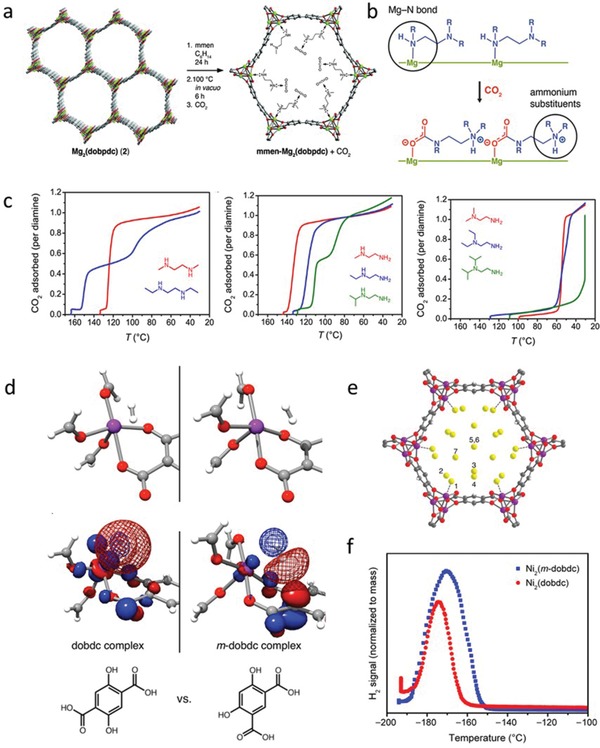
a) Postsynthetic modification scheme for diamine‐functionalized MOF‐74 analogue. Reproduced with permission.[qv: 102a] Copyright 2012, American Chemical Society. b) Scheme for carbamate chain formation during CO_2_ chemisorption on diamine functionalized Mg_2_(dobpdc). c) CO_2_ adsorption step tuning by varying the structure of diamines. b,c) Reproduced with permission.[qv: 103c] Copyright 2017, American Chemical Society. d) Calculated binding modes of H_2_ in Co(*p‐*dobdc) and Co(*m*‐dobdc) complexes. Reproduced with permission.^106^ Copyright 2014, American Chemical Society. e) H_2_/D_2_ binding sites in Co(*m*‐dobdc). f) Differences in temperature‐programmed desorption of H_2_ in Ni_2_(*m*‐dobdc) and Ni_2_(dobdc) indicating the different interaction energies. e,f) Reproduced with permission.^107^ Copyright 2018, American Chemical Society.

As an acidic and polarizable gas, capture of CO_2_ may be affected by several distinct mechanisms. The capture of CO_2_ can take place at various stages of the power generation process, utilizing precombustion flue gas or postcombustion flue gas as feed in order to reduce the net release of CO_2_. Furthermore, the design of sorbents for direct air capture (DAC) is desired to decrease the concentration of existing CO_2_ in the atmosphere.^99^ The differing feed compositions and operating conditions (in particular temperature and pressure) imposes different requirements vis. uptake capacity and selectivity on the materials. The incumbent technology for scrubbing postcombustion flue gas (≈15% CO_2_ and ≈75% N_2_ at ≈1 bar) utilizes chemical absorption by aqueous alkanolamine solutions.^100^ The current benchmark adsorbent for adsorptive CO_2_ capture is Mg‐MOF‐74 with a CO_2_ uptake of 5.28 mmol g^‐1^ under dry simulated conditions. The primary functionality of the MOF originates from the presence of open metal sites lining the porous channels. The exceptional CO_2_ uptake of Mg‐MOF‐74 results from the combination of low atomic weight of the Mg element as well as increased ionic character of Mg—O bond, which endowed the framework with high low‐pressure affinity.[qv: 15c,67a] Despite the remarkable performance, the relevance of open metal site‐based sorbents for CO_2_ capture has been impeded by passivation of the guest binding sites by water, limiting their use in post‐combustion capture.^101^ To address this problem, Long's group and others have turned to diamine grafting in isoreticular frameworks to leverage the highly selective chemisorption mechanism (**Figure** **13**a).^102^ Optimization of the available pore space may be achieved by extending the scaffolding linker or reducing the chain length of the grafted diamine (Figure 13b,c).^103^ For instance, expanded MOF‐74 structure possessing 18.4 Å wide channels lined with open metal coordination sites was obtained via ligand extension. Further functionalization of the expanded MOF‐74 structure with *N,N'*‐dimethylethylenediamine resulted in enhanced gas diffusion and extraordinary CO_2_ adsorption properties, indicating the key role of pore space and functionalization.[qv: 102a] The case study of MOF‐74 in CO_2_ capture highlights the efficacy of rational tuning in versatile platforms to yield favorable application performance. When subject to a similar approach of atomic‐level design, the MOF‐74 family has displayed promise in hydrocarbon separations.^104^ The importance of selectivity in separation‐based applications typically imposes stringent requirements on the MOF scaffold and attaining the “appropriate” pore system, which may even outweigh functionality considerations. The recent successful examples have evidenced a shift toward “ultra‐tuning” or “fine‐tuning” approaches to meet these requirements. For size‐sieving based separations, the design of the framework geometry—, e.g., triangular apertures in **fcu** MOFs—enables the attenuation of large stepwise changes in the linear dimension of the organic linkers into smaller variation of the aperture sizes.^105^ Eddaoudi's group, for example, adopted isoreticular chemistry to synthesize rare‐earth metal (yttrium and terbium) and fumarate ligand‐based fcu‐MOF with a specific window size that can afford sieving of branched paraffins (isopentane, isobutane) from normal paraffins (n‐pentane, n‐butane). Deliberately regulated window aperture size (≈4.7 Å) endowed the structure with molecular sieving property to exclude larger‐sized branched paraffins whilst capturing the smaller‐sized normal paraffins.^105^ Interpenetration of self‐dual networks is another strategy, which is effective to control pore size with greater precision.[qv: 37c]

Adsorption‐based storage of energy‐related gases, namely hydrogen and methane, faces lofty performance targets to attain in order to meet the demand for on‐board vehicular storage. For H_2_, the 2020 U.S. Department of Energy target is 4.5 wt% gravimetric capacity, 30 g L^−1^ volumetric capacity under a maximum delivery pressure of 100 atm between ‐40 to 60 °C. Kapelewski et al. reported a MOF‐74 isomer using 4,6‐dioxido‐1,3‐benzenedicarboxylate which increased the charge density at the OMSs, leading to record hydrogen binding enthalpies (Figure 13d).^106^ The nickel‐based material possessed volumetric capacity of 11.0 g L^−1^ at ambient condition (Figure 13e,f).^107^ For CH_4_, 263 cm^3^ cm^‐3^ volumetric capacity has been stipulated. Peng et al. established in 2013 that HKUST‐1 MOF was able to meet the latter target provided ideal packing efficiency was retained.^108^ Recently, Fairen‐Jimenez and co‐workers reported a protocol for the preparation of HKUST‐1 monoliths capable of near‐ideal packing densities.^109^ The methane uptake in the best‐performing sorbents is largely governed by van der Waals interactions, which suggests scope for design for porosity optimization. However, an operational caveat is the 5 bar minimum pressure for desorption, which negates the CH_4_ uptake at the low pressure region of the isotherm.^108^ The utilization of methane‐induced phase transitions to address this issue was demonstrated using Fe and Co benzenedipyrazolate frameworks.^110^ Kundu et al. investigated the methane storage and delivery characteristics of flexible MIL‐53‐type MOFs, which are functionalized by incorporating hydroxyl groups to the linker to control structural phase transition.^111^ Stepwise methane sorption isotherms of MIL‐53(Al)‐OH confirmed gradual transformation of narrow pores to large pore as a function of methane pressure. It exhibited a deliverable methane capacity of 164 v/v at 65 bar and 298 K and performed better than MIL‐53(Al)‐OH_2_ due to π–π stacking control over the breathing behavior. This unique structure–property relationship shows that the installation of functional moieties to control structural breathing transitions may be promising for the development of more effective materials.

### 5.2 Water Harvesting

**Figure 14:**
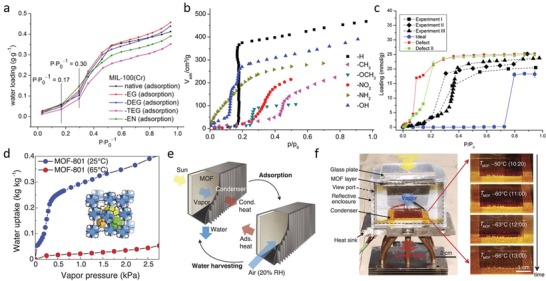
a) Water adsorption isotherms of MIL‐100 (Cr) and EG‐, DEG‐, TEG‐ and EN‐grafted MIL‐100(Cr) at 25 °C. Reproduced with permission.[qv: 7a] Copyright 2013, Elsevier. b) Water adsorption isotherms of CAU‐10‐X (X: —H, —NO_2_, —NH_2_, —CH_3_, —OCH_3_, and —OH) at 25 °C. Reproduced with permission.[qv: 7b] Copyright 2013, American Chemical Society. c) Water adsorption isotherms for UiO‐66 with ideal unit cell and defective unit cells obtained from simulations at 25 °C along with the experimental results. Reproduced with permission.^118^ Copyright 2014, The Royal Society of Chemistry. d) Water adsorption isotherms of MOF‐801 at temperatures of 25 °C and 65 °C. The inset shows the crystal structure of MOF‐801. Each large sphere indicates a different cavity. Black, C; red, O; blue polyhedra, Zr. e) Schematic and f) proof‐of‐concept water‐harvesting device. d–f) Reproduced with permission.[qv: 116b] Copyright 2017, The American Association for the Advancement of Science.

As the latest Global Risks Report published by the World Economic Forum (2019) states, the water crisis is among the top five risks of greatest concern in terms of impact, and it is perceived as more likely than many other economic, environmental, geopolitical, social, and technological risks.^112^ Notably, fresh water accounts for only 2.5% of the total water stocks, and a large percentage of it (68.7%) is locked in icebergs, posing a challenge in accessibility and feasibility for producing potable fresh water.^113^ Moreover, considering the uneven spatial distribution of stocks (e.g., landlocked and isolated areas), contamination in readily available resources and effect of temporal fluctuations on water supply‐demand, it was reported that two thirds of the world population experience fresh water scarcity at least one month in a year and half a billion people deal with water shortage throughout the year.^114^ Atmosphere is an alternative source of fresh water, holding about 0.13 quintillion (10^18^) liters of water in form of moisture/water droplet.^113^ Presently, fog and dew water collection are the main atmospheric water harvesting (AWH) technologies proposed to alleviate the water stresses.^115^ Fog water collection is a viable approach, in which water carried by the wind is trapped on fine mesh and naturally drips into a container, yet presence of correct wind speeds and 100% relative humidity (RH) are essential for this operation. Similarly, dew water collection allows liquid water harvesting from atmosphere via passive radiative condensers, which is limited by daily climate variation‐dependent dew formation conditions. Very recently, adsorption‐based water harvesting has been proposed for regions where fog and dew water collection are not feasible to operate due to weather conditions.[qv: 42a,116] It requires a sorbent material with remarkable uptake capacity to capture moisture at a relatively high humidity level and low temperature during nighttime, and regenerate during daytime by utilizing the naturally available solar‐thermal energy and/or waste heat. Although zeolites and silica are conventional and economic porous sorbents, their exploitation is hindered by the low water‐uptake and/or elevated regeneration temperatures ascribed to the strong water–sorbent interactions. In this respect, MOFs are alternative candidates with diverse water sorption characteristics, high porosities, and modular reticular chemistry. Atomic‐ and molecular‐level design have been the key to fulfill the essential hydrolytic stability prerequisite for durable AWH operation.^117^ Susceptibility of MOFs for degradation over ligand displacement and hydrolysis has paved the way for regulating kinetic and thermodynamic factors by means of engineering the electronic and steric effects of the metal and ligand.^117^ Moreover, water sorption characteristics of MOFs have been tailored by in situ functional linkers substitution, postsynthetic hydrophilic group grafting and defect generation for favorable water adsorption/desorption processes.^7^ For example, Wickenheisser et al. demonstrated that it is possible to increase water uptake capacities and shift water adsorption isotherms to lower relative pressures by grafting hydrophilic groups (EG (ethylene glycol), DEG (diethylene glycol), TEG (triethylene glycol), and EN (ethylenediamine)) onto coordinatively unsaturated metal sites in MIL‐100(Cr) (**Figure** **14**a).[qv: 7a] Likewise, CAU‐10 series bearing functional groups with different polarity and size exhibited distinct water sorption behaviors (Figure 14b).[qv: 7b] By systematically tuning the synthesis conditions, it is possible to create defects in form of missing‐linker defects and missing‐cluster defects in pristine MOF structures, particularly in Zr‐MOFs (e.g., UiO‐66, UiO‐67, UiO‐68, MOF‐801) by a virtue of exceptional stabilities.[qv: 7c] Complimentary molecular simulations suggested that missing organic linkers in UiO‐66 increase the hydrophilicity and improves the water affinity (Figure 14c).^118^ Rieth et al. reported that presence of unsaturated metal sites ensures reversible and continuous pore filling in large 1D channels of Co_2_Cl_2_(BTDD) MOF (22 Å), which matches to the critical diameter (*D*_c_) for irreversible capillary condensation, since expeditious preadsorption of water molecules on unsaturated metal sites narrows down the pore size below *D*_c_.[qv: 116a] Notably, Co_2_Cl_2_(BTDD) was tested under simulated daytime–nighttime desert conditions (daytime values of 45 °C and 5% RH, nighttime values of 25 °C and 35% RH) to investigate ideal deliverable water capacity. An ideal deliverable water capacity of 0.82 kg water per kg of Co_2_Cl_2_(BTDD) was predicted based on the characteristic curve‐derived isotherms under representative conditions. Moreover, Co_2_Cl_2_(BTDD) was predicted to release 0.87 L of water per kg MOF per cycle when operated in a RH region between 65% (nighttime, 25 °C) and 10% (daytime, 66 °C). Importantly, the first proof‐of‐concept of such a system demonstrated in 2017 reported encouraging findings for harvesting water from air using a sorbent material.[qv: 116b] 0.30 L of water was estimated to be potentially harvested using 1 kg of MOF‐801 in a single cycle relying on nighttime humid air (65% RH, 25 °C) and daytime ambient sunlight (10% RH, 66 °C) (Figure 14d–f). Moreover, in arid regions (5–40% RH, 10–40 °C), natural fluctuation of weather conditions was able to drive water‐harvesting process with high‐energy efficiency, resulting in collection of 0.1 kg of water per kg of MOF‐801 leveraging the available photothermal energy and passive natural cooling.[qv: 116d]

### 5.3 Water Remediation

The previous section has addressed the direct capture of water from the atmosphere to meet the growing demand for potable water. A complementary approach is the removal of contaminants of water, including heavy metal cations, dyes, agrochemicals, and other organic pollutants. This is necessary not only to mitigate threats to human health arising from poor quality water sources, but also to limit long‐term impact on the Earth's ecosystems. The modularity of MOFs is a distinct advantage allowing tailoring of performance to accommodate the distinct chemical characters of the contaminants and pollutants. Several reviews have been published summarizing the development of MOF adsorbents in water remediation.^119^ Other provide in‐depth consideration of certain classes of pollutants including heavy and radioactive metals,[qv: 53e,120] hazardous organics,^121^ as well as pharmaceuticals and personal care products.^122^

A prerequisite for MOFs being applied to water remediation is a sufficient stability to survive prolonged immersion in aqueous environments. This is a more stringent requirement compared to the case of direct water capture, where liquid water with low ionic content is involved. When removing charged compounds, for example, dyes like methylene blue and methyl orange, the pH is highly relevant in determining the separation performance. In general, in line with hard/soft acid/base (HSAB) theory, the combination of high‐valence metal cations with carboxylate‐based ligands is a route to derive chemically stable materials.^123^ However, for water remediation, more stringent stability criteria are typically required. Bezverkhyy et al. reported drift toward acidic pH when MIL‐100(Fe) and MIL‐53(Fe) were dispersed in deionized water solutions.^124^ When the pH was maintained at 7 by base titration, collapse of the frameworks occurred. The observation exposes the need for more careful consideration of the MOF structural properties in order to avoid undesired structural degradation and leaching problems.

From the design perspective, a few synthetic trends have been identified to address threats to stability, in particular media with high acidity, basicity, or concentrations of metal‐coordinating ligands. Increasing the connectivity of the organic ligands is an effective way to improve the stability of the MOF structures. PCN‐250, incorporating a tetratopic carboxylate ligand, is stable in aqueous solutions ranging from pH 1 to pH 11.^125^ The aluminum chain‐based 467‐MOF, incorporating elongated tritopic ligands, is stable within the same pH range up to 36 h.^126^ BUT‐12/BUT‐13, also based on tritopic ligands and zirconium clusters is stable from pH 0 to pH 10.^127^ The improved stability of these MOFs increases their applicability in water remediation. For example, the fluorescent property of the ligands in BUT‐12/BUT‐13 enabled the use of the frameworks for simultaneous detection and capture of antibiotics and nitroaromatics in aqueous environments.

The presence of strongly coordinating anions, such as F^−^, SO_4_^2−^, and PO_4_^3−^, requires alternative strategies to be developed.^128^ Besides hard‐acid/hard‐base metal‐linker combinations, the pairing of soft acids with soft bases may also generate stable frameworks less susceptible to attack by hard Lewis base anions. The PCN‐602 framework bearing the [Ni_8_(OH)_4_(H_2_O)_2_Pz_12_] cluster was shown to withstand KF, K_3_PO_4,_ and Na_2_CO_3_ aqueous solutions, albeit with lower acid tolerance (up to pH 4).^128^ Alternative coordination motifs, such as phenolates and phosphonates, have also been shown to yield ultrastable porous materials. Devic et al. reported the MIL‐163 structure based on a phenolate‐based ligand, which was able to survive exposure to 0.1 M phosphate‐buffered‐saline (PBS) media.^129^ Wang and co‐workers reported three zirconium phosphonate frameworks with tetrahedral‐shaped linkers, denoted SZ‐1 to SZ‐3, exhibiting remarkable stabilities in fuming acid conditions.^130^ The latter compound showed hitherto unprecedented stability in aqua regia and good uranyl uptake behavior. The combined porosity and stability of these compounds is promising for harsher applications such as nuclear waste remediation.

Due to the multiplicity of contaminants of interest for adsorptive separation, it is challenging to presume general design strategies for MOF adsorbents. Regardless, a promising structural motif worth mentioning is the hydroxyl/aqua capped sites in several high‐valence metal‐based MOFs, which may emerge from defect engineering or topological reasons. The capacity of these sites for interaction with both metal ions and coordinating ligands suggests their use in adsorptive removal of contaminants. The defect sites in Zr MOFs have proven to be effective in the removal of heavy or toxic metals. Li and co‐workers reported the capture of As(V) from aqueous solution by UiO‐66 with a high uptake capacity of 303 mg g^−1^, which was attributed in part to the hydroxyl groups in the framework.^131^ Farha's group have tested selenate and selenite removal in a series of zirconium MOFs.^132^ The uptake capacities possessed clear dependence on the number of defect sites (i.e., not occupied by structural ligands). The capture of pertechnetate (TcO_4_^−^) and perrhenate (ReO_4_^−^) was shown to occur by a pseudo‐ion exchange mechanism at the hydroxyl or water‐capped sites in the Zr node of NU‐1000 by the same group.^133^ As an example of the second case (capture of coordinating organic ligands), Gu et al. reported a superior uptake capacity of the organophosphorous pesticides, glyphosate, and glufosinate, by UiO‐67(Zr).^134^ The phosphate groups were postulated to interact with the nodes given the high affinity of phosphate for zirconium.

The amenability for atomic‐level design in MOF materials is envisaged to produce high‐performance adsorbents in water remediation. Meanwhile, ongoing development toward understanding the adsorption mechanisms and optimizing the scalable preparation of MOF materials is required to enhance the viability of MOFs for this application.

## 6 Summary and Outlook

We have summarized the strategies and recent progress on atomic‐ and molecular‐design of MOFs and MOFs derivatives to develop advanced functional materials for broad applications such as gas storage, gas separation, water harvesting, water remediation, HER, OER, ORR, CO_2_RR, and rechargeable batteries. The following design strategies are presented to prepare functional pristine MOFs and MOFs derivatives for energy and environmental applications. 1) Rational selection and coordination of the MOFs' primary constituents, and functional group and defect introduction into MOFs can be employed to render pristine MOFs with unique structural properties and chemical functionalities. Application‐targeted modulated MOFs have exhibited promising performance in environmental applications such as gas storage/separation and water harvesting/remediation as well as energy conversion applications, including OER and CO_2_RR. 2) Carbon materials derived from MOFs can be endowed with exceptional electronic and structural properties through N‐doping. Graphitization degree, nitrogen‐dopant content, dopant configuration, surface area, and morphology of the carbon structure can be modified by engineering the pristine MOFs and tuning the posttreatment conditions. Moreover, carbons with defective lattice can be prepared by designing N‐ and low boiling point metal‐containing MOFs. Importantly, single metal atom sites can be created in the carbons by designing MOFs via ionic coordination, pore confinement, N‐posttreatment and NPs‐to‐single atom conversion strategies. 3) Transformation of MOFs into transition metal atom incorporated MOF‐derivatives (transition metal oxides, chalcogenides, pnictides, and nitrides) via a prior pristine MOF design offers unprecedented electronic structures and porous morphologies beneficial for mass and electron transfer. Notably, carbons and transition metal‐based materials derived from atomic‐ and molecular‐level designed MOFs outperform the state‐of‐the‐art materials in energy conversion and storage fields such as electrocatalysis (OER, ORR, HER, CO_2_RR) and rechargeable batteries as discussed through recent case studies.

Although considerable progress has been made in application‐directed design of MOFs and MOFs derivatives, some shortcomings and challenges affecting their efficacy still exist. 1) Despite the great versatility of MBBs and modularity of MOFs, only several well‐known MOFs such as ZIF‐67, ZIF‐8, PBAs, and MILs are commonly employed to transform MOFs into MOFs derivatives. However, precise control of reticular chemistry could guide preparation of diverse MOFs derivatives, facilitating exploration of functional materials with novel physicochemical properties, optimal chemical composition and abundant electrochemical active sites. In pursuit of this, PSM can be an effective approach to realize such pristine MOFs. 2) Treatment and characterization of ordered heterogeneity by the introduction of functionalities into defined sites is well established. However, other strategies to attain diversity, e.g., the generation of hybrid MOF phases by mixed‐component syntheses, require deeper understanding of distribution. This is important for the rationalization of “synergistic” effects and deviations from simple mixing systems. 3) Lack of profound insight into the control of loading content and coordination environment of atomically dispersed metal species in MOF‐derived carbons has been a typical challenge for preparation of highly active single‐atom sites. To accomplish this goal, strategies can be developed to prepare bimetallic pristine MOFs with precise metallic content adjustment, which are prone to homogeneously lose one metallic component and stabilize the other through intrinsically formed defects during post‐treatment. In addition, although the top‐down NPs‐to‐single atom conversion is more controllable compared to bottom‐up MOF‐to‐single atom approaches, only a few types of single metal atoms have been reported (Ni, Pd, Au, Pt) so far. Thus, this strategy offers an avenue for controlled preparation of diverse single metallic atom sites. Moreover, advanced in situ and operando characterization techniques coupled with theoretical investigations should be employed to realize the single‐atom formation and coordination processes. The same techniques can be used to unveil structure–performance relationship and catalytic mechanism. 4) Low conductivity of pristine MOFs has been a significant concern for their effective utilization in energy storage and conversion fields. Thus, more efforts should be devoted toward atomic‐ and molecular‐design of MOFs to advance the through‐bond and through‐space approaches[qv: 79a] for improving the intrinsic charge mobility. 5) Despite the formation of local and extended defects in MOFs and MOFs derivatives, controllable synthesis of defects remains as a challenge, especially for MOF‐derived carbons. Predesigning pristine MOFs with controllable content of volatile elements that can progressively distract the carbon lattice is believed to offer great opportunities for defect engineering. Moreover, advanced material characterization tools and theoretical calculations are required to elucidate the interplay between defect concentration/type and activity. 6) For gas storage and sorption applications, the geometry and pore environment play important roles in the performance. Rational design strategies for the adjustment of porosity with higher precision are necessary to attain even higher performances. 7) Passive atmospheric water harvesting is still in its infancy, necessitating rational synthesis of MOFs through atomic‐ and molecular‐design strategies such as proper linker‐metal node choice and defect generation to satisfy the prerequisites of essential hydrolytic stability and high water adsorption uptake/delivery capacity under desired conditions. In addition, effect of chemical functionalities and structural properties on water adsorption kinetics and mechanism needs to be explored to gain in depth understanding on the structure–activity relationship.

To sum up, application‐targeted atomic‐ and molecular‐level design of MOFs has been a central approach to improve the performance of MOFs and MOF‐derivatives. Modularity of MOFs' surface chemistry, thanks to versatility of the molecular building blocks, has offered boundless structures with unique functionalities and morphologies for a wide range of energy (e.g., CO_2_RR, ORR, OER, HER, rechargeable batteries) and environmental (e.g., gas storage/separation, water harvesting/remediation) applications. Although it is clear that MOFs provide vast possibilities for developing functional materials through atomic‐ and molecular‐level design, aforementioned challenges hinder further enhancement of existing materials' performance and expansion of material choice with target functionalities. Unprecedented potential of as‐designed MOFs and MOF‐derivatives in energy and environmental fields is believed to be encouraging for future efforts to overcome the current challenges. Particularly, design guidelines toward specific applications should be proposed by gaining in‐depth understanding on structure/functionality–activity relationship through complimentary advanced material characterizations and theoretical calculations.

## Conflict of Interest

The authors declare no conflict of interest.

## References

[1] a) P. Z. Moghadam , A. Li , S. B. Wiggin , A. Tao , A. G. Maloney , P. A. Wood , S. C. Ward , D. Fairen‐Jimenez , Chem. Mater. 2017, 29, 2618;

[2] a) N. W. Ockwig , O. Delgado‐Friedrichs , M. O'Keeffe , O. M. Yaghi , Acc. Chem. Res. 2005, 38, 176; 15766236 10.1021/ar020022l

[3] H. Furukawa , J. Kim , N. W. Ockwig , M. O'Keeffe , O. M. Yaghi , J. Am. Chem. Soc. 2008, 130, 11650. 18693690 10.1021/ja803783c

[4] a) O. K. Farha , I. Eryazici , N. C. Jeong , B. G. Hauser , C. E. Wilmer , A. A. Sarjeant , R. Q. Snurr , S. T. Nguyen , A. Ö. Yazaydın , J. T. Hupp , J. Am. Chem. Soc. 2012, 134, 15016; 22906112 10.1021/ja3055639

[5] a) S. Ma , H.‐C. Zhou , Chem. Commun. 2010, 46, 44; 10.1039/c0cc03448g 20936245

[6] a) D. Zhao , D. J. Timmons , D. Yuan , H.‐C. Zhou , Acc. Chem. Res. 2010, 44, 123; 21126015 10.1021/ar100112y

[7] a) M. Wickenheisser , F. Jeremias , S. K. Henninger , C. Janiak , Inorg. Chim. Acta 2013, 407, 145;

[8] a) Y. Zhao , Z. Song , X. Li , Q. Sun , N. Cheng , S. Lawes , X. Sun , Energy Storage Mater. 2016, 2, 35;

[9] a) S. M. Cohen , Chem. Rev. 2011, 112, 970; 21916418 10.1021/cr200179u

[10] a) S. Dissegna , K. Epp , W. R. Heinz , G. Kieslich , R. A. Fischer , Adv. Mater. 2018, 30, 1704501; 10.1002/adma.201704501 29363822

[11] a) J. Tang , Y. Yamauchi , Nat. Chem. 2016, 8, 638; 10.1038/nchem.2548 27325086

[12] a) L. Zhang , Q. Xu , J. Niu , Z. Xia , Phys. Chem. Chem. Phys. 2015, 17, 16733; 26033301 10.1039/c5cp02014j

[13] D. J. Tranchemontagne , J. L. Mendoza‐Cortés , M. O'Keeffe , O. M. Yaghi , Chem. Soc. Rev. 2009, 38, 1257. 19384437 10.1039/b817735j

[14] a) N. L. Rosi , J. Kim , M. Eddaoudi , B. Chen , M. O'Keeffe , O. M. Yaghi , J. Am. Chem. Soc. 2005, 127, 1504; 15686384 10.1021/ja045123o

[15] a) P. D. C. Dietzel , Y. Morita , R. Blom , H. Fjellvåg , Angew. Chem., Int. Ed. 2005, 44, 6354; 10.1002/anie.200501508 16145702

[16] a) J. H. Cavka , S. Jakobsen , U. Olsbye , N. Guillou , C. Lamberti , S. Bordiga , K. P. Lillerud , J. Am. Chem. Soc. 2008, 130, 13850; 18817383 10.1021/ja8057953

[17] a) M. D. Allendorf , C. A. Bauer , R. K. Bhakta , R. J. T. Houk , Chem. Soc. Rev. 2009, 38, 1330; 19384441 10.1039/b802352m

[18] R. Luebke , Y. Belmabkhout , Ł. J. Weseliński , A. J. Cairns , M. Alkordi , G. Norton , Ł. Wojtas , K. Adil , M. Eddaoudi , Chem. Sci. 2015, 6, 4095. 29218176 10.1039/c5sc00614g PMC5707464

[19] a) G. Férey , C. Serre , C. Mellot‐Draznieks , F. Millange , S. Surblé , J. Dutour , I. Margiolaki , Angew. Chem., Int. Ed. 2004, 43, 6296; 10.1002/anie.200460592 15372634

[20] M. Lammert , S. Bernt , F. Vermoortele , D. E. De Vos , N. Stock , Inorg. Chem. 2013, 52, 8521. 23829498 10.1021/ic4005328

[21] a) C. K. Brozek , L. Bellarosa , T. Soejima , T. V. Clark , N. López , M. Dincă , Chem. Eur. J. 2014, 20, 6871; 24782420 10.1002/chem.201402682

[22] M. Eddaoudi , J. Kim , N. Rosi , D. Vodak , J. Wachter , M. O'Keeffe , O. M. Yaghi , Science 2002, 295, 469. 11799235 10.1126/science.1067208

[23] P. Li , Q. Chen , T. C. Wang , N. A. Vermeulen , B. L. Mehdi , A. Dohnalkova , N. D. Browning , D. Shen , R. Anderson , D. A. Gómez‐Gualdrón , F. M. Cetin , J. Jagiello , A. M. Asiri , J. F. Stoddart , O. K. Farha , Chem 2018, 4, 1022.

[24] J. Lyu , X. Zhang , K.‐i. Otake , X. Wang , P. Li , Z. Li , Z. Chen , Y. Zhang , M. C. Wasson , Y. Yang , P. Bai , X. Guo , T. Islamoglu , O. K. Farha , Chem. Sci. 2019, 10, 1186. 30774917 10.1039/c8sc04220a PMC6349059

[25] H. Wang , X. Dong , J. Lin , S. J. Teat , S. Jensen , J. Cure , E. V. Alexandrov , Q. Xia , K. Tan , Q. Wang , D. H. Olson , D. M. Proserpio , Y. J. Chabal , T. Thonhauser , J. Sun , Y. Han , J. Li , Nat. Commun. 2018, 9, 1745. 29717138 10.1038/s41467-018-04152-5 PMC5931593

[26] V. Guillerm , T. Grancha , I. Imaz , J. Juanhuix , D. Maspoch , J. Am. Chem. Soc. 2018, 140, 10153. 30064215 10.1021/jacs.8b07050

[27] M. B. Chambers , X. Wang , L. Ellezam , O. Ersen , M. Fontecave , C. Sanchez , L. Rozes , C. Mellot‐Draznieks , J. Am. Chem. Soc. 2017, 139, 8222. 28535334 10.1021/jacs.7b02186

[28] a) T. Zhou , Y. Du , A. Borgna , J. Hong , Y. Wang , J. Han , W. Zhang , R. Xu , Energy Environ. Sci. 2013, 6, 3229;

[29] a) R. G. Pearson , J. Am. Chem. Soc. 1963, 85, 3533;

[30] S. Wang , M. Wahiduzzaman , L. Davis , A. Tissot , W. Shepard , J. Marrot , C. Martineau‐Corcos , D. Hamdane , G. Maurin , S. Devautour‐Vinot , Nat. Commun. 2018, 9, 4937. 30467390 10.1038/s41467-018-07414-4 PMC6250719

[31] a) Z. Wang , S. M. Cohen , Chem. Soc. Rev. 2009, 38, 1315; 19384440 10.1039/b802258p

[32] O. K. Farha , K. L. Mulfort , J. T. Hupp , Inorg. Chem. 2008, 47, 10223. 18928272 10.1021/ic8018452

[33] a) Y. K. Hwang , D.‐Y. Hong , J.‐S. Chang , S. H. Jhung , Y.‐K. Seo , J. Kim , A. Vimont , M. Daturi , C. Serre , G. Férey , Angew. Chem., Int. Ed. 2008, 47, 4144; 10.1002/anie.200705998 18435442

[34] a) J. Jiang , F. Gándara , Y.‐B. Zhang , K. Na , O. M. Yaghi , W. G. Klemperer , J. Am. Chem. Soc. 2014, 136, 12844; 25157587 10.1021/ja507119n

[35] a) S. Yuan , W. Lu , Y.‐P. Chen , Q. Zhang , T.‐F. Liu , D. Feng , X. Wang , J. Qin , H.‐C. Zhou , J. Am. Chem. Soc. 2015, 137, 3177; 25714137 10.1021/ja512762r

[36] D. N. Bunck , W. R. Dichtel , Chem. ‐ Eur. J. 2013, 19, 818.23280516

[37] a) S. Subramanian , M. J. Zaworotko , Angew. Chem., Int. Ed. 1995, 34, 2127;

[38] P. Nugent , V. Rhodus , T. Pham , B. Tudor , K. Forrest , L. Wojtas , B. Space , M. Zaworotko , Chem. Commun. 2013, 49, 1606. 10.1039/c3cc37695h 23340547

[39] a) A. Cadiau , K. Adil , P. M. Bhatt , Y. Belmabkhout , M. Eddaoudi , Science 2016, 353, 137; 27387945 10.1126/science.aaf6323

[40] a) H. Li , C. E. Davis , T. L. Groy , D. G. Kelley , O. M. Yaghi , J. Am. Chem. Soc. 1998, 120, 2186;

[41] P. D. C. Dietzel , R. E. Johnsen , R. Blom , H. Fjellvåg , Chem. Eur. J. 2008, 14, 2389. 18203217 10.1002/chem.200701370

[42] a) H. Furukawa , F. Gándara , Y.‐B. Zhang , J. Jiang , W. L. Queen , M. R. Hudson , O. M. Yaghi , J. Am. Chem. Soc. 2014, 136, 4369; 24588307 10.1021/ja500330a

[43] a) V. Bon , V. Senkovskyy , I. Senkovska , S. Kaskel , Chem. Commun. 2012, 48, 8407; 10.1039/c2cc34246d 22801712

[44] a) K. Gong , F. Du , Z. Xia , M. Durstock , L. Dai , Science 2009, 323, 760; 19197058 10.1126/science.1168049

[45] a) X.‐F. Li , K.‐Y. Lian , L. Liu , Y. Wu , Q. Qiu , J. Jiang , M. Deng , Y. Luo , Sci. Rep. 2016, 6, 23495; 27002190 10.1038/srep23495 PMC4802320

[46] a) H. Kim , K. Lee , S. I. Woo , Y. Jung , Phys. Chem. Chem. Phys. 2011, 13, 17505; 21946759 10.1039/c1cp21665a

[47] a) D. Hulicova‐Jurcakova , M. Seredych , G. Q. Lu , T. J. Bandosz , Adv. Funct. Mater. 2009, 19, 438;

[48] a) S. H. Ahn , M. J. Klein , A. Manthiram , Adv. Energy Mater. 2017, 7, 1601979;

[49] W. Chaikittisilp , M. Hu , H. Wang , H.‐S. Huang , T. Fujita , K. C.‐W. Wu , L.‐C. Chen , Y. Yamauchi , K. Ariga , Chem. Commun. 2012, 48, 7259. 10.1039/c2cc33433j 22710974

[50] Z. Chen , R. Wu , Y. Liu , Y. Ha , Y. Guo , D. Sun , M. Liu , F. Fang , Adv. Mater. 2018, 30, 1802011. 10.1002/adma.201802011 29888482

[51] a) J.‐W. Jeon , R. Sharma , P. Meduri , B. W. Arey , H. T. Schaef , J. L. Lutkenhaus , J. P. Lemmon , P. K. Thallapally , M. I. Nandasiri , B. P. McGrail , ACS Appl. Mater. Interfaces 2014, 6, 7214; 24784542 10.1021/am500339x

[52] G. Yilmaz , C. F. Tan , M. Hong , G. W. Ho , Adv. Funct. Mater. 2018, 28, 1704177.

[53] a) L. Ye , G. Chai , Z. Wen , Adv. Funct. Mater. 2017, 27, 1606190;

[54] a) G. Yilmaz , C. F. Tan , Y. F. Lim , G. W. Ho , Adv. Energy Mater. 2019, 9, 1802983;

[55] M. H. Naveen , K. Shim , M. S. A. Hossain , J. H. Kim , Y. B. Shim , Adv. Energy Mater. 2017, 7, 1602002.

[56] a) X.‐F. Yang , A. Wang , B. Qiao , J. Li , J. Liu , T. Zhang , Acc. Chem. Res. 2013, 46, 1740; 23815772 10.1021/ar300361m

[57] a) P. Hu , Z. Huang , Z. Amghouz , M. Makkee , F. Xu , F. Kapteijn , A. Dikhtiarenko , Y. Chen , X. Gu , X. Tang , Angew. Chem. 2014, 126, 3486; 10.1002/anie.201309248 24599751

[58] a) Y. Lei , F. Mehmood , S. Lee , J. Greeley , B. Lee , S. Seifert , R. E. Winans , J. W. Elam , R. J. Meyer , P. C. Redfern , Science 2010, 328, 224; 20378815 10.1126/science.1185200

[59] a) C. Marichy , M. Bechelany , N. Pinna , Adv. Mater. 2012, 24, 1017; 22278762 10.1002/adma.201104129

[60] a) H. Wei , X. Liu , A. Wang , L. Zhang , B. Qiao , X. Yang , Y. Huang , S. Miao , J. Liu , T. Zhang , Nat. Commun. 2014, 5, 5634; 25465918 10.1038/ncomms6634

[61] a) P. Yin , T. Yao , Y. Wu , L. Zheng , Y. Lin , W. Liu , H. Ju , J. Zhu , X. Hong , Z. Deng , Angew. Chem., Int. Ed. 2016, 55, 10800; 10.1002/anie.201604802 27491018

[62] L. Jiao , G. Wan , R. Zhang , H. Zhou , S.‐H. Yu , H.‐L. Jiang , Angew. Chem., Int. Ed. Engl. 2018, 57, 8525. 29742316 10.1002/anie.201803262

[63] a) T. He , S. Chen , B. Ni , Y. Gong , Z. Wu , L. Song , L. Gu , W. Hu , X. Wang , Angew. Chem. 2018, 130, 3551; 10.1002/anie.201800817 29380509

[64] a) Y. Chen , S. Ji , Y. Wang , J. Dong , W. Chen , Z. Li , R. Shen , L. Zheng , Z. Zhuang , D. Wang , Angew. Chem., Int. Ed. 2017, 56, 6937; 10.1002/anie.201702473 28402604

[65] F. Li , G.‐F. Han , H.‐J. Noh , S.‐J. Kim , Y. Lu , H. Y. Jeong , Z. Fu , J.‐B. Baek , Energy Environ. Sci. 2018, 11, 2263.

[66] a) L. Fan , P. F. Liu , X. Yan , L. Gu , Z. Z. Yang , H. G. Yang , S. Qiu , X. Yao , Nat. Commun. 2016, 7, 10667; 26861684 10.1038/ncomms10667 PMC4749971

[67] a) D. Britt , H. Furukawa , B. Wang , T. G. Glover , O. M. Yaghi , Proc. Natl. Acad. Sci. USA 2009, 106, 20637; 19948967 10.1073/pnas.0909718106 PMC2791636

[68] a) B. Weng , C. R. Grice , W. Meng , L. Guan , F. Xu , Y. Yu , C. Wang , D. Zhao , Y. Yan , ACS Energy Lett. 2018, 3, 1434;

[69] a) J. Su , Y. Yang , G. Xia , J. Chen , P. Jiang , Q. Chen , Nat. Commun. 2017, 8, 14969; 28440269 10.1038/ncomms14969 PMC5413983

[70] a) S. Lim , K. Suh , Y. Kim , M. Yoon , H. Park , D. N. Dybtsev , K. Kim , Chem. Commun. 2012, 48, 7447; 10.1039/c2cc33439a 22729073

[71] a) Y. Li , J. Lu , ACS Energy Lett. 2017, 2, 1370;

[72] a) B. Y. Guan , X. Y. Yu , H. B. Wu , X. W. Lou , Adv. Mater. 2017, 29, 1703614; 10.1002/adma.201703614 28960488

[73] a) J. A. Turner , Science 2004, 305, 972; 15310892 10.1126/science.1103197

[74] a) J. K. Nørskov , T. Bligaard , A. Logadottir , J. Kitchin , J. G. Chen , S. Pandelov , U. Stimming , J. Electrochem. Soc. 2005, 152, J23;

[75] Y. Zheng , Y. Jiao , Y. Zhu , L. H. Li , Y. Han , Y. Chen , M. Jaroniec , S.‐Z. Qiao , J. Am. Chem. Soc. 2016, 138, 16174. 27960327 10.1021/jacs.6b11291

[76] a) Y. Ge , P. Dong , S. R. Craig , P. M. Ajayan , M. Ye , J. Shen , Adv. Energy Mater. 2018, 8, 1800484;

[77] a) Y. Wang , J. Mao , X. Meng , L. Yu , D. Deng , X. Bao , Chem. Rev. 2019, 119, 1806; 30575386 10.1021/acs.chemrev.8b00501

[78] a) A. J. Clough , J. W. Yoo , M. H. Mecklenburg , S. C. Marinescu , J. Am. Chem. Soc. 2014, 137, 118; 25525864 10.1021/ja5116937

[79] a) L. Sun , M. G. Campbell , M. Dincă , Angew. Chem., Int. Ed. 2016, 55, 3566; 10.1002/anie.201506219 26749063

[80] a) I. C. Man , H. Y. Su , F. Calle‐Vallejo , H. A. Hansen , J. I. Martínez , N. G. Inoglu , J. Kitchin , T. F. Jaramillo , J. K. Nørskov , J. Rossmeisl , ChemCatChem 2011, 3, 1159;

[81] a) Z. Xue , Y. Li , Y. Zhang , W. Geng , B. Jia , J. Tang , S. Bao , H. P. Wang , Y. Fan , Z. W. Wei , Adv. Energy Mater. 2018, 8, 1801564;

[82] Y. Wang , K. S. Chen , J. Mishler , S. C. Cho , X. C. Adroher , Appl. Energy 2011, 88, 981.

[83] J. Spendelow , D. Papageorgopoulos , Fuel Cells 2011, 11, 775.

[84] S. Kattel , G. Wang , J. Mater. Chem. A 2013, 1, 10790.

[85] Z. S. Wu , L. Chen , J. Liu , K. Parvez , H. Liang , J. Shu , H. Sachdev , R. Graf , X. Feng , K. Müllen , Adv. Mater. 2014, 26, 1450. 24293313 10.1002/adma.201304147

[86] Y. Deng , B. Chi , J. Li , G. Wang , L. Zheng , X. Shi , Z. Cui , L. Du , S. Liao , K. Zang , Adv. Energy Mater. 2019, 9, 1802856.

[87] J. Li , M. Chen , D. A. Cullen , S. Hwang , M. Wang , B. Li , K. Liu , S. Karakalos , M. Lucero , H. Zhang , C. Lei , H. Xu , G. E. Sterbinsky , Z. Feng , D. Su , K. L. More , G. Wang , Z. Wang , G. Wu , Nat. Catal. 2018, 1, 935.

[88] a) N. Kornienko , Y. Zhao , C. S. Kley , C. Zhu , D. Kim , S. Lin , C. J. Chang , O. M. Yaghi , P. Yang , J. Am. Chem. Soc. 2015, 137, 14129; 26509213 10.1021/jacs.5b08212

[89] a) X. Jiang , H. Li , J. Xiao , D. Gao , R. Si , F. Yang , Y. Li , G. Wang , X. Bao , Nano Energy 2018, 52, 345;

[90] F. Pan , H. Zhang , K. Liu , D. Cullen , K. More , M. Wang , Z. Feng , G. Wang , G. Wu , Y. Li , ACS Catal. 2018, 8, 3116.

[91] Z. R. Jiang , H. Wang , Y. Hu , J. Lu , H. L. Jiang , ChemSusChem 2015, 8, 878. 25651098 10.1002/cssc.201403230

[92] a) Q. Yang , C. C. Yang , C. H. Lin , H. L. Jiang , Angew. Chem., Int. Ed. 2019, 58, 3511; 10.1002/anie.201813494 30569535

[93] a) J. Liang , Y.‐B. Huang , R. Cao , Coord. Chem. Rev. 2019, 378, 32;

[94] a) B. Zhang , C. M. Ghimbeu , C. Laberty , C. Vix‐Guterl , J. M. Tarascon , Adv. Energy Mater. 2016, 6, 1501588;

[95] L. Wang , Y. Han , X. Feng , J. Zhou , P. Qi , B. Wang , Coord. Chem. Rev. 2016, 307, 361.

[96] a) L.‐L. Wu , Z. Wang , Y. Long , J. Li , Y. Liu , Q.‐S. Wang , X. Wang , S.‐Y. Song , X. Liu , H.‐J. Zhang , Small 2017, 13, 1604270; 10.1002/smll.201604270 28244189

[97] a) B. Li , H.‐M. Wen , W. Zhou , B. Chen , J. Phys. Chem. Lett. 2014, 5, 3468; 26278595 10.1021/jz501586e

[98] a) Z. R. Herm , E. D. Bloch , J. R. Long , Chem. Mater. 2014, 26, 323;

[99] Y. Lin , C. Kong , Q. Zhang , L. Chen , Adv. Energy Mater. 2017, 7, 1601296.

[100] G. T. Rochelle , Science 2009, 325, 1652. 19779188 10.1126/science.1176731

[101] J. A. Mason , T. M. McDonald , T.‐H. Bae , J. E. Bachman , K. Sumida , J. J. Dutton , S. S. Kaye , J. R. Long , J. Am. Chem. Soc. 2015, 137, 4787. 25844924 10.1021/jacs.5b00838

[102] a) T. M. McDonald , W. R. Lee , J. A. Mason , B. M. Wiers , C. S. Hong , J. R. Long , J. Am. Chem. Soc. 2012, 134, 7056; 22475173 10.1021/ja300034j

[103] a) P. J. Milner , J. D. Martell , R. L. Siegelman , D. Gygi , S. C. Weston , J. R. Long , Chem. Sci. 2018, 9, 160; 29629084 10.1039/c7sc04266c PMC5869309

[104] a) J. E. Bachman , M. T. Kapelewski , D. A. Reed , M. I. Gonzalez , J. R. Long , J. Am. Chem. Soc. 2017, 139, 15363; 28981259 10.1021/jacs.7b06397

[105] A. H. Assen , Y. Belmabkhout , K. Adil , P. M. Bhatt , D.‐X. Xue , H. Jiang , M. Eddaoudi , Angew. Chem., Int. Ed. 2015, 54, 14353. 10.1002/anie.201506345 26429515

[106] M. T. Kapelewski , S. J. Geier , M. R. Hudson , D. Stück , J. A. Mason , J. N. Nelson , D. J. Xiao , Z. Hulvey , E. Gilmour , S. A. FitzGerald , M. Head‐Gordon , C. M. Brown , J. R. Long , J. Am. Chem. Soc. 2014, 136, 12119. 25130365 10.1021/ja506230r

[107] M. T. Kapelewski , T. Runčevski , J. D. Tarver , H. Z. H. Jiang , K. E. Hurst , P. A. Parilla , A. Ayala , T. Gennett , S. A. FitzGerald , C. M. Brown , J. R. Long , Chem. Mater. 2018, 30, 8179. 10.1021/acs.chemmater.8b03276 PMC7067217 32165787

[108] Y. Peng , V. Krungleviciute , I. Eryazici , J. T. Hupp , O. K. Farha , T. Yildirim , J. Am. Chem. Soc. 2013, 135, 11887. 23841800 10.1021/ja4045289

[109] T. Tian , Z. Zeng , D. Vulpe , M. E. Casco , G. Divitini , P. A. Midgley , J. Silvestre‐Albero , J.‐C. Tan , P. Z. Moghadam , D. Fairen‐Jimenez , Nat. Mater. 2017, 17, 174. 29251723 10.1038/nmat5050

[110] J. A. Mason , J. Oktawiec , M. K. Taylor , M. R. Hudson , J. Rodriguez , J. E. Bachman , M. I. Gonzalez , A. Cervellino , A. Guagliardi , C. M. Brown , P. L. Llewellyn , N. Masciocchi , J. R. Long , Nature 2015, 527, 357. 26503057 10.1038/nature15732

[111] a) A. Boutin , M.‐A. Springuel‐Huet , A. Nossov , A. Gédéon , T. Loiseau , C. Volkringer , G. Férey , F.‐X. Coudert , A. H. Fuchs , Angew. Chem., Int. Ed. 2009, 48, 8314; 10.1002/anie.200903153 19780084

[112] W. E. Forum , The Global Risks Report 2019, 14th Edition 2019, 1, https://www.weforum.org/reports/the-global-risks-report-2019, (accessed: April 2019).

[113] P. H. Gleick , Water in Crisis: A Guide to the World's Fresh Water Resources, Oxford University Press, Oxford 1993.

[114] M. M. Mekonnen , A. Y. Hoekstra , Sci. Adv. 2016, 2, e1500323. 26933676 10.1126/sciadv.1500323 PMC4758739

[115] a) M. Fessehaye , S. A. Abdul‐Wahab , M. J. Savage , T. Kohler , T. Gherezghiher , H. Hurni , Renewable Sustainable Energy Rev. 2014, 29, 52;

[116] a) A. J. Rieth , S. Yang , E. N. Wang , M. Dincă , ACS Cent. Sci. 2017, 3, 668; 28691080 10.1021/acscentsci.7b00186 PMC5492259

[117] J. Canivet , A. Fateeva , Y. Guo , B. Coasne , D. Farrusseng , Chem. Soc. Rev. 2014, 43, 5594. 24875439 10.1039/c4cs00078a

[118] P. Ghosh , Y. J. Colón , R. Q. Snurr , Chem. Commun. 2014, 50, 11329. 10.1039/c4cc04945d 25116653

[119] a) N. A. Khan , Z. Hasan , S. H. Jhung , J. Hazard. Mater. 2013, 244, 444; 23195596 10.1016/j.jhazmat.2012.11.011

[120] P. A. Kobielska , A. J. Howarth , O. K. Farha , S. Nayak , Coord. Chem. Rev. 2018, 358, 92.

[121] a) Z. Hasan , S. H. Jhung , J. Hazard. Mater. 2015, 283, 329; 25305363 10.1016/j.jhazmat.2014.09.046

[122] Y. Xu , T. Liu , Y. Zhang , F. Ge , R. M. Steel , L. Sun , J. Mater. Chem. A 2017, 5, 12001.

[123] S. Yuan , L. Feng , K. Wang , J. Pang , M. Bosch , C. Lollar , Y. Sun , J. Qin , X. Yang , P. Zhang , Adv. Mater. 2018, 30, 1704303. 10.1002/adma.201704303 29430732

[124] I. Bezverkhyy , G. Weber , J.‐P. Bellat , Microporous Mesoporous Mater. 2016, 219, 117.

[125] D. Feng , K. Wang , Z. Wei , Y.‐P. Chen , C. M. Simon , R. K. Arvapally , R. L. Martin , M. Bosch , T.‐F. Liu , S. Fordham , Nat. Commun. 2014, 5, 5723. 25474702 10.1038/ncomms6723

[126] Z. W. Wang , M. Chen , C. S. Liu , X. Wang , H. Zhao , M. Du , Chem.: Eur. J. 2015, 21, 17215. 26449991 10.1002/chem.201502615

[127] B. Wang , X.‐L. Lv , D. Feng , L.‐H. Xie , J. Zhang , M. Li , Y. Xie , J.‐R. Li , H.‐C. Zhou , J. Am. Chem. Soc. 2016, 138, 6204. 27090616 10.1021/jacs.6b01663

[128] X.‐L. Lv , K. Wang , B. Wang , J. Su , X. Zou , Y. Xie , J.‐R. Li , H.‐C. Zhou , J. Am. Chem. Soc. 2016, 139, 211. 27936748 10.1021/jacs.6b09463

[129] G. Mouchaham , L. Cooper , N. Guillou , C. Martineau , E. Elkaïm , S. Bourrelly , P. L. Llewellyn , C. Allain , G. Clavier , C. Serre , T. Devic , Angew. Chem., Int. Ed. 2015, 54, 13297. 10.1002/anie.201507058 26457412

[130] T. Zheng , Z. Yang , D. Gui , Z. Liu , X. Wang , X. Dai , S. Liu , L. Zhang , Y. Gao , L. Chen , Nat. Commun. 2017, 8, 15369. 28555656 10.1038/ncomms15369 PMC5459948

[131] C. Wang , X. Liu , J. P. Chen , K. Li , Sci. Rep. 2015, 5, 16613. 26559001 10.1038/srep16613 PMC4642326

[132] a) A. J. Howarth , M. J. Katz , T. C. Wang , A. E. Platero‐Prats , K. W. Chapman , J. T. Hupp , O. K. Farha , J. Am. Chem. Soc. 2015, 137, 7488; 26000611 10.1021/jacs.5b03904

[133] R. J. Drout , K. Otake , A. J. Howarth , T. Islamoglu , L. Zhu , C. Xiao , S. Wang , O. K. Farha , Chem. Mater. 2018, 30, 1277.

[134] X. Zhu , B. Li , J. Yang , Y. Li , W. Zhao , J. Shi , J. Gu , ACS Appl. Mater. Interfaces 2014, 7, 223. 25514633 10.1021/am5059074

[135] J. Tang , R. R. Salunkhe , H. Zhang , V. Malgras , T. Ahamad , S. M. Alshehri , N. Kobayashi , S. Tominaka , Y. Ide , J. H. Kim , Sci. Rep. 2016, 6, 30295. 27471193 10.1038/srep30295 PMC4965863

[136] H.‐L. Jiang , Q. Yang , C.‐C. Yang , C.‐H. Lin , Angew. Chem., Int. Ed. 2019, 58, 3511. 10.1002/anie.201813494 30569535

[137] Y. Qu , Z. Li , W. Chen , Y. Lin , T. Yuan , Z. Yang , C. Zhao , J. Wang , C. Zhao , X. Wang , Nat. Catal. 2018, 1, 781.

